# Systematic discovery of drug interaction mechanisms

**DOI:** 10.15252/msb.20156098

**Published:** 2015-04-29

**Authors:** Guillaume Chevereau, Tobias Bollenbach

**Affiliations:** IST AustriaKlosterneuburg, Austria

**Keywords:** antibiotics, drug combination design, drug interaction mechanisms, *Escherichia coli*, general principles of biological systems

## Abstract

Drug combinations are increasingly important in disease treatments, for combating drug resistance, and for elucidating fundamental relationships in cell physiology. When drugs are combined, their individual effects on cells may be amplified or weakened. Such drug interactions are crucial for treatment efficacy, but their underlying mechanisms remain largely unknown. To uncover the causes of drug interactions, we developed a systematic approach based on precise quantification of the individual and joint effects of antibiotics on growth of genome-wide *Escherichia coli* gene deletion strains. We found that drug interactions between antibiotics representing the main modes of action are highly robust to genetic perturbation. This robustness is encapsulated in a general principle of bacterial growth, which enables the quantitative prediction of mutant growth rates under drug combinations. Rare violations of this principle exposed recurring cellular functions controlling drug interactions. In particular, we found that polysaccharide and ATP synthesis control multiple drug interactions with previously unexplained mechanisms, and small molecule adjuvants targeting these functions synthetically reshape drug interactions in predictable ways. These results provide a new conceptual framework for the design of multidrug combinations and suggest that there are universal mechanisms at the heart of most drug interactions.

## Introduction

**Figure 1:**
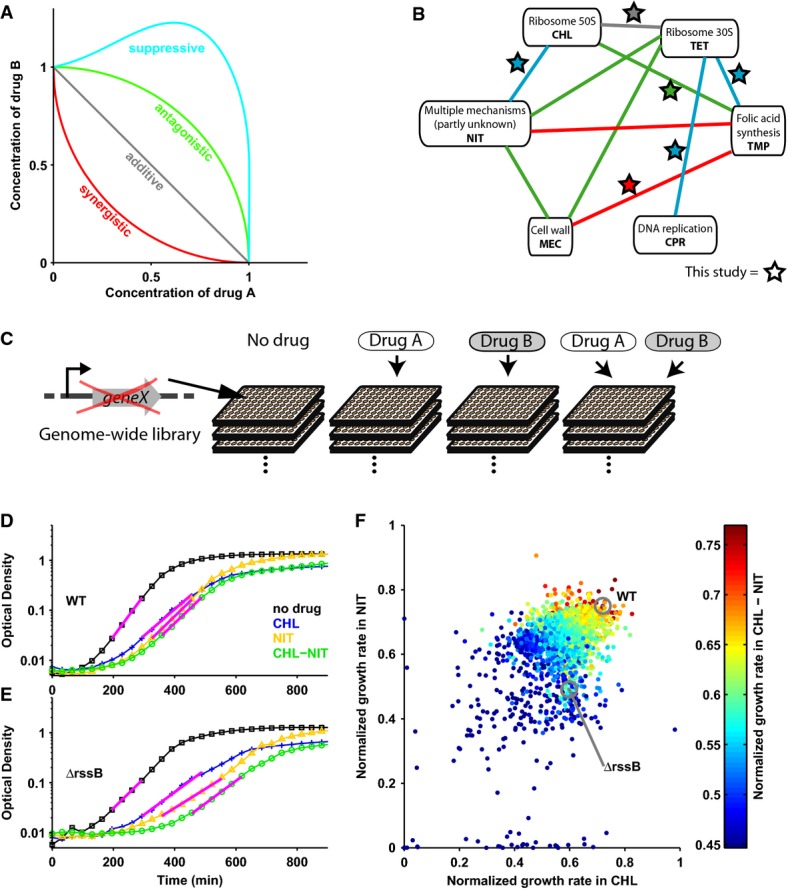
Systematically identifying genes that affect drug interactions

Drugs play a crucial role in elucidating fundamental relationships in cell physiology (Falconer *et al*, 2011). When drugs are combined, interactions like synergism and antagonism can occur (Loewe, 1928; Keith *et al*, 2005) (Fig1A); such drug interactions are often critical for the success of multidrug treatments (Pillai *et al*, 2005) and can slow or accelerate antibiotic resistance evolution (Chait *et al*, 2007; Hegreness *et al*, 2008). A case in point is the synergistic combination of trimethoprim and sulfa drugs which has been applied successfully for decades (Pillai *et al*, 2005) even though the mechanism of synergism has long remained elusive (Nichols *et al*, 2011). Synergism and antagonism occur frequently between antimicrobials and are largely determined by the primary cellular target of the drugs that are combined (Yeh *et al*, 2006; Ocampo *et al*, 2014). However, synergistic drug interactions are rarely explained by the genetic interactions between the corresponding drug target genes (Cokol *et al*, 2011). To design combinations exploiting the full potential of existing drugs, a deeper understanding of the underlying mechanisms of drug interactions is urgently needed. Due to the vast number of possible drug combinations, general principles that are valid across diverse drug pairs could greatly facilitate the identification of drug interaction mechanisms. Recent work in this direction revealed scaling laws describing the effects of resistance mutations on drug interactions (Chait *et al*, 2007; Wood *et al*, 2014); further, the effects of three or more drugs appear largely predictable from the two-drug effects (Wood *et al*, 2012). However, the underlying mechanisms of most two-drug interactions remain unknown.

Schematic: lines of equal growth (isoboles) in two-dimensional concentration space of drugs A and B; isobole shape determines drug interaction (Loewe, 1928). Synergistic drug pairs have stronger than additive, antagonistic ones weaker than additive effect on growth. In suppressive interactions, the combination effect is weaker than that of one of the drugs alone.

Interaction network for six antibiotics: synergism is red; antagonism, green; suppression, blue; stars show drug combinations investigated here.

Schematic illustrating approach, see the text for details.

Wild-type *E. coli* (WT) growth curves without drug (black), under chloramphenicol (blue), nitrofurantoin (yellow), and chloramphenicol–nitrofurantoin combination (green); magenta lines are exponential fits (Materials and Methods).

As (D) for the *rssB* mutant which has a lower growth rate but unchanged drug interaction.

Scatterplot: growth rates of ˜4,000 *E. coli* gene deletion mutants under chloramphenicol and nitrofurantoin alone and under the combination (color scale).

Drug interactions could be caused by physicochemical effects, for example when one drug simply enhances the permeability of the cell envelope for another (Jawetz & Gunnison, 1953); alternatively, they may have more complex causes, specifically if one drug triggers a regulatory response, which affects the action of another. While many genes affect the cell's sensitivity to individual drugs (Hillenmeyer *et al*, 2008; Nichols *et al*, 2011) and recent work suggested that certain drug resistance mutations may affect drug interactions (Munck *et al*, 2014; Wood *et al*, 2014; Rodriguez de Evgrafov *et al*, 2015), it is unclear to what extent genetic perturbations can alter drug interactions. Likewise, the cellular functions that control these interactions are largely unknown. To pinpoint their underlying causes, we developed a systematic approach for identifying genes that reshape drug interactions: using precise growth rate measurements of a genome-wide set of *E. coli* gene deletion mutants (Baba *et al*, 2006), we show that drug interactions are robust to most, but not all, genetic changes. We present a general principle encapsulating this robustness, which enables the quantitative prediction of mutant growth under drug combinations. Rare mutants violating this principle expose cellular functions governing each drug interaction. We establish that diverse drug interactions are recurrently controlled by central cellular functions, in particular polysaccharide synthesis and ATP synthesis.

## Results and Discussion

We began by quantifying the growth rates of ∼4,000 nonessential *E. coli* gene deletion mutants (Baba *et al*, 2006) under six different antibiotic pairs and their constituent individual drugs (Materials and Methods). We selected antibiotics with diverse modes of action (Table1) and drug pairs covering all interaction types (Fig1A and B). Drug concentrations were adjusted to inhibit wild-type growth under individual drugs by ∼30%; the same concentrations were used when drugs were combined, leading to different levels of growth inhibition (Materials and Methods). In total, we measured over 50,000 growth curves (optical density increase over time) using a dedicated robotic system (Fig1C–F; Materials and Methods). These growth rate measurements were highly reproducible (Supplementary Fig S1), and consistent with established changes in mutant sensitivity to individual antibiotics (Tamae *et al*, 2008; Liu *et al*, 2010; Nichols *et al*, 2011): for example, DNA repair mutants were sensitive to ciprofloxacin. Drugs with related mode of action had similar effects on growth of genome-wide mutants (e.g., Pearson correlation ρ = 0.68 for chloramphenicol and tetracycline), and the effects of drug combinations were usually most similar to those of the constituent drugs (Supplementary Fig S2; Materials and Methods). These observations confirmed our expectation that perturbations of cell physiology caused by drug combinations are mostly an overlay of effects caused by the constituent drugs.

**Table 1:** Antibiotics used in this study

Abbreviation	Drug	Mode of action (known target)	Concentration
CHL	Chloramphenicol	Protein synthesis (50S ribosome subunit)	1 µg/ml
CPR	Ciprofloxacin	DNA replication (gyrase)	4 ng/ml
MEC	Mecillinam	Cell wall (Penicillin Binding Protein)	38 ng/ml
NIT	Nitrofurantoin	Multiple mechanisms	2 µg/ml
TET	Tetracycline	Protein synthesis (30S ribosome subunit)	150 ng/ml
TMP	Trimethoprim	Folic acid synthesis (DHFR)	80 ng/ml

**Figure 2:**
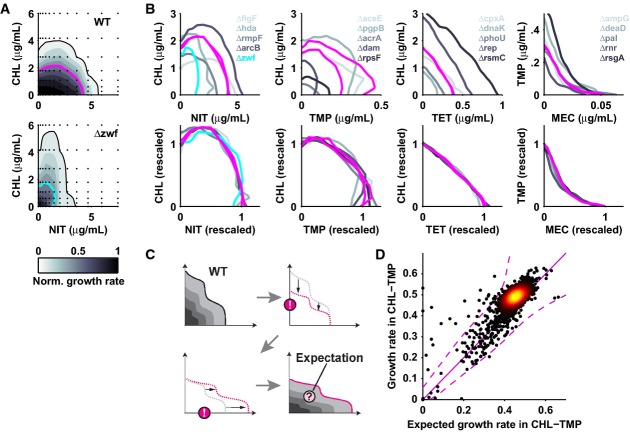
Drug interactions do not change for most gene deletions, enabling the quantitative prediction of mutant growth rates under drug combinations

We next identified a general principle characterizing drug interactions in mutants. We hypothesized that the shape of the two-drug growth response surface *g*(*a*,*b*), which defines the interaction (Fig1A), does not change qualitatively in most mutants (here, *g* denotes growth rate and *a*, *b* the drug concentrations). To test this hypothesis, we measured 108 mutant response surfaces in two-dimensional drug concentration matrices covering different drug pairs; this selection included mutants with strongly altered sensitivity to the individual drugs (Materials and Methods). We found that the vast majority of mutant response surfaces *g*^mut^(*a*,*b*) were well approximated by a linearly rescaled wild-type surface: *g*^mut^(*a*,*b*) =γ*g*^WT^(*αa*,*βb*) with scaling factors for maximum growth rate *γ* and for drug concentrations *α*, *β* (Fig2A and B; Supplementary Figs S3 and S4). The sensitivity to one or both of the drugs often changed considerably, yet the response surface shape was generally preserved (Fig2A); these observations held for all drug pairs and for mutants affecting diverse cellular functions (Fig2B; Supplementary Fig S4), suggesting that most genetic perturbations do not affect drug interactions.

Growth of WT (top) and *zwf* mutant (bottom) across two-dimensional chloramphenicol–nitrofurantoin concentration space; IC_50_ lines are magenta (WT) and cyan (*zwf*).

Top row: IC_50_ lines of WT and mutants for antibiotic combinations with different drug interactions: chloramphenicol–nitrofurantoin (suppressive), chloramphenicol–trimethoprim (antagonistic), chloramphenicol–tetracycline (additive), trimethoprim–mecillinam (synergistic). Bottom row: IC_50_ lines upon concentration rescaling (see text). While sensitivity to individual drugs changes in mutants, the drug interaction does not (see also Supplementary Figs S3 and S4). Magenta lines show WT.

Schematic: calculation of expected mutant growth rates under drug combinations assuming concentration rescaling as in (A, B) (Materials and Methods).

Scatterplot: measured versus expected mutant growth rates under chloramphenicol–trimethoprim; identity line is in solid magenta; dashed magenta lines show 95% confidence interval (Materials and Methods).

To test whether this conservation of drug interactions holds generally, we devised a strategy for predicting genome-wide mutant growth responses to drug combinations. For all ∼4,000 mutants, we calculated the expected response to the drug combination by first rescaling the wild-type response surface according to the individual drug responses measured at fixed concentrations. At the rescaled drug concentrations, we then used the interaction coefficient of the wild-type, which quantifies the response to the drug combination relative to the Bliss additive expectation (Yeh *et al*, 2006), to calculate each mutant's expected growth rate under the drug combination (Fig2C; Materials and Methods). The central assumption of this procedure is that the drug interaction is universally invariant, that is it is the same in mutants as in the wild-type upon rescaling of the drug concentrations. For all drug pairs and the vast majority of mutants, the growth rates measured at fixed concentration of the drug combination (Fig1C–F) faithfully followed this prediction (Fig2D; Supplementary Fig S5). These observations thus revealed a general principle of bacterial growth under drug combinations, which encapsulates the high robustness of drug interactions to genetic perturbations and enables the quantitative prediction of mutant growth rates under drug combinations.

**Figure 3:**
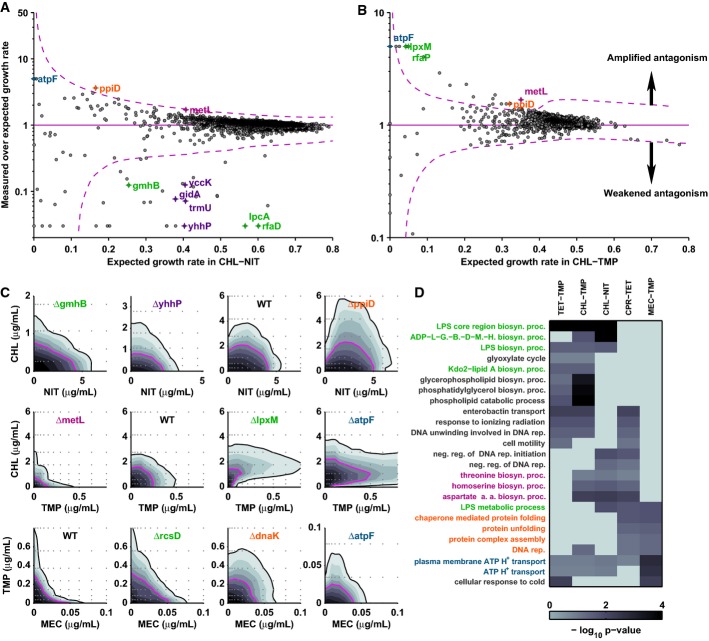
Drug interactions are controlled by a confined set of recurring cellular functions

The identification of this general principle empowered us to pinpoint ‘outlier’ mutants with unexpected growth response to drug combinations. These outliers are of key interest as they could have altered drug interaction; together with functional information on the mutated gene, they can thus point at the underlying drug interaction mechanism. Clear outliers for which the observed growth rate under the drug combination (Fig1F) deviated significantly from the expected growth rate were rare (typically < 1% of mutants; Fig2D; Supplementary Fig S5), facilitating this investigation. We measured the response surface of the strongest outliers for each drug combination in fine resolution 12 × 8 concentration matrices (Materials and Methods). For each drug pair, we thus identified several mutants with clearly reshaped drug interaction (Fig3, Supplementary Fig S6). Drug interactions were often weakened or removed in these mutants; they were also amplified in certain mutants and, in some cases, entirely new ‘synthetic’ drug interactions appeared (Fig3C). In a thiamin synthesis hypomorph, chloramphenicol–trimethoprim even became reciprocally suppressive; that is, addition of chloramphenicol on top of trimethoprim increased growth and vice versa (Supplementary Fig S7). We further observed clear biases in interaction changes: chloramphenicol–nitrofurantoin suppression was weakened or entirely removed in most mutants affecting this interaction (Fig3A); in contrast, chloramphenicol–trimethoprim antagonism was often amplified to suppression (Fig3B and C; Supplementary Fig S6L–Q), while other drug combinations showed more balanced interaction changes in both directions (Supplementary Fig S5). These data show that different drug combinations have varying potential for changing their drug interaction type, even if the wild-type interaction is similar. Interestingly, for the additive chloramphenicol–tetracycline combination, none of the outlier mutants showed any change in drug interaction: while the sensitivity to the constituent drugs often changed, additivity was generally preserved (Supplementary Fig S3). This observation is consistent with previous results that this interaction changed little in strains that had evolved resistance to chloramphenicol–tetracycline (Munck *et al*, 2014). Thus, the chloramphenicol–tetracycline drug interaction appears robust to genetic perturbations. Together, these data show that most drug interactions can be removed, amplified, and even qualitatively changed to a different interaction type by rare genetic perturbations, while other interactions are robust.

Scatterplot: deviation from expectation of measured mutant growth rates under chloramphenicol–nitrofurantoin versus expected growth rate; dashed magenta lines show 95% confidence interval (Materials and Methods); mutants above this region have amplified antagonism, mutants below weakened antagonism.

As (A) for chloramphenicol–trimethoprim.

Growth of mutants in two-dimensional concentration gradients of chloramphenicol–nitrofurantoin (top), chloramphenicol–trimethoprim (middle), and trimethoprim–mecillinam (bottom). Outlier mutants have altered drug interactions in agreement with the results shown in (A, B). Drug interactions change in polysaccharide synthesis (*gmhB*, *lpxM*, *rcsD*; green), ATP synthesis (*atpF*; blue), chaperoning (*dnaK*, *ppiD*; orange), and amino acid synthesis (*metL*; magenta) mutants; see also Supplementary Fig S6.

Clustergram showing gene ontology terms enriched among outliers for multiple drug pairs and corresponding *P*-values (Materials and Methods, Supplementary Table S1).

We next identified cellular functions controlling drug interactions; among these, the synthesis of secreted polysaccharides (capsular and lipopolysaccharides, LPS) and ATP synthesis stood out in that they affected virtually all drug interactions. Firstly, manual inspection of the functional annotation of the outlier mutants (Keseler *et al*, 2005) for each drug pair, and systematic gene ontology enrichment analysis congruently exposed specific functions controlling the respective interaction (Fig3D, Supplementary Table S1, Materials and Methods): for example, perturbing tRNA processing consistently removed chloramphenicol–nitrofurantoin suppression (Fig3A and C; Supplementary Fig S6X and Y); similarly, ribosome production and assembly altered ciprofloxacin–tetracycline suppression (*dksA*, *rsgA*, *ksgA* in Supplementary Fig S5C; Supplementary Table S1), confirming previous results (Bollenbach *et al*, 2009). Secondly, we noticed that certain functions recurrently control multiple drug interactions. Besides polysaccharide and ATP synthesis (discussed below), chaperone deletions (*ppiD*, *dnaK*) consistently caused amplified or synthetic suppression for distinct drug pairs and perturbing amino acid synthesis (*metL*) amplified chloramphenicol–nitrofurantoin suppression but removed trimethoprim–chloramphenicol antagonism (Fig3C, Supplementary Fig S6). Thus, multiple drug interactions are controlled by few recurring cellular functions.

**Figure 4:**
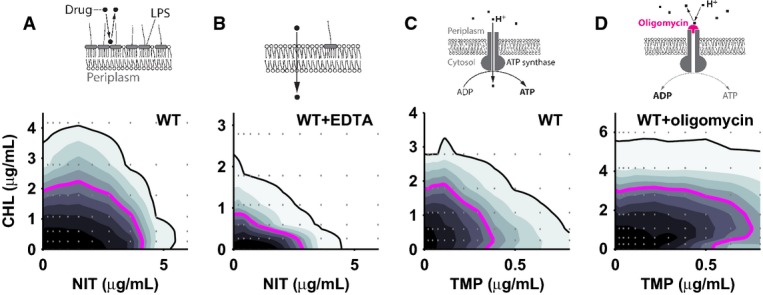
Targeted reshaping of drug interactions using small molecule adjuvants

Polysaccharide synthesis affects the majority of drug interactions (Fig3C and D; Supplementary Fig S6): perturbing this function removed chloramphenicol–nitrofurantoin suppression and trimethoprim–mecillinam synergy (*gmhB*, *rcsD* in Fig3C; *lpcA* in Supplementary Fig S6); in contrast, it led to synthetic suppression between trimethoprim and chloramphenicol (*lpxM* in Fig3C; Supplementary Fig S6M and N). Polysaccharide synthesis mutants have modified outer membrane composition, which affects the uptake of molecules dependent on their chemical properties (Nikaido & Vaara, 1985). Hence, a plausible cause of these drug interactions is that bacteria regulate polysaccharide synthesis in response to certain antibiotics, which then affects the uptake of other drugs. Consistent with this mechanism, antibiotics are known to affect polysaccharide synthesis (Rothfield & Pearlman-Kothencz, 1969) and LPS synthesis mutants have increased sensitivity to chloramphenicol (Fig3C; Supplementary Fig S6V); thus, stimulation of LPS synthesis by nitrofurantoin could explain chloramphenicol–nitrofurantoin suppression. To directly test this scenario, we removed outer membrane LPS using ethylenediaminetetraacetic acid (EDTA) (Nikaido & Vaara, 1985). Strikingly, LPS removal increased sensitivity to chloramphenicol, abolished chloramphenicol–nitrofurantoin suppression, and rendered this drug interaction purely additive (Fig4A and B). Together, these data support that regulated changes in cell envelope composition, which affect drug uptake, are a recurring mechanism underlying chloramphenicol–nitrofurantoin suppression and other antibiotic interactions.

A, B Growth of WT in chloramphenicol–nitrofurantoin concentration gradient in the absence (A) and in the presence of EDTA at 2 mM (B); MIC lines are black, IC_50_ lines magenta. EDTA addition removes the suppressive drug interaction between chloramphenicol and nitrofurantoin and increases sensitivity to chloramphenicol. Schematics: effect of EDTA on outer membrane LPS composition and drug uptake.

C, D As (A) for chloramphenicol–trimethoprim in the absence (C) and in the presence of oligomycin at 160 μg/ml (D). Oligomycin addition changes the drug interaction from antagonistic to suppressive. Schematics: oligomycin effect on ATP synthase.

ATP synthesis also controls multiple drug interactions (Fig3C and D). Specifically, the ATP synthase mutant *atpF* was more sensitive to trimethoprim and this sensitivity was reduced by chloramphenicol or mecillinam, leading to suppression (Fig3C); a similar effect occurred for ciprofloxacin–tetracycline (Supplementary Fig S6S). A thiamin synthesis hypomorph, which also has perturbed energy metabolism, behaved similarly (Supplementary Fig S7). Further, ATP synthase expression increased two-fold in response to trimethoprim (Supplementary Fig S8), suggesting that cells respond homeostatically to ATP deficiency. To test independently whether ATP synthesis affects drug interactions, we specifically blocked the proton pore of the ATP synthase F_O_ subunit using drugs (oligomycin, venturicidin; Materials and Methods). Indeed, inhibiting ATP synthase led to suppression between trimethoprim and chloramphenicol (Fig4C and D; Supplementary Fig S9), supporting that imbalances in energy metabolism cause this synthetic drug interaction. Together, these data suggest a mechanistic scenario in which impaired ATP synthase function leads to decreased intracellular ATP levels, which may become growth-limiting in the presence of trimethoprim or ciprofloxacin; concurrent translation inhibition by chloramphenicol would reduce global ATP turnover, replenish the intracellular ATP pool (Schneider *et al*, 2002), and thus lead to increased growth, which would explain the observed suppressive interaction. No suppression would occur in the wild-type where ATP is likely in excess and not growth-limiting. At a molecular level, decreased ATP concentrations might limit growth by aggravating DNA repair and synthesis (Waldstein *et al*, 1974), which is likely the growth-limiting process in the presence of drugs targeting DNA synthesis; however, other ATP-dependent processes could also contribute to suppression. Overall, our results show that perturbations of central cellular functions, unrelated to the common antibiotic targets, can reshape diverse drug interactions.

We established a general principle of bacterial growth, which enables the prediction of mutant growth rates under drug combinations from their growth under the individual drugs alone (Fig2, Supplementary Fig S5). This principle may hold more generally and should be tested for combinations of other challenges such as osmotic, temperature, or pH stress in future work. While conceptually similar empirical laws are an integral part of physics, they are still scarce in biology (Scott & Hwa, 2011). Even without understanding their molecular origins, such principles are powerful since they enable the prediction of quantitative phenotypes. Here, such a principle was crucial for systematically revealing antibiotic interaction mechanisms.

The identification of cellular functions controlling drug interactions offers new strategies for the rational design of multidrug combinations. Specifically, we identified targets for potential adjuvants, which could reshape antibiotic interactions: thiamin synthesis inhibitors could render the chloramphenicol–trimethoprim combination reciprocally suppressive (Supplementary Fig S7); such reciprocal suppression may slow down resistance evolution but is extremely scarce among natural antibiotic interactions (Chait *et al*, 2007). LPS synthesis inhibitors could remove chloramphenicol–nitrofurantoin suppression (Fig4A and B), thus preserving advantages of an untapped drug combination while increasing its potency. Drugs inhibiting cellular functions that control antibiotic interactions (LPS synthesis, ATP synthesis, and chaperoning; Fig3C and D) are in development (Moreau *et al*, 2008; Evans *et al*, 2010; Du *et al*, 2011; Balemans *et al*, 2012). These inhibitors could reshape drug interactions even if they have poor antimicrobial activity alone since most mutants we identified have only mild growth defects. Finally, our approach revealed that certain drug combinations are robust to mutations (Supplementary Fig S3) or change primarily toward weakened antagonism (Fig3A). The origins of such robustness and biases in drug interaction changes are unknown. Still, such insights can be used to avoid loss of synergism due to mutations occurring in treatments, which is a serious concern (Pena-Miller *et al*, 2013; Munck *et al*, 2014). It will be exciting to extend the systematic approach presented here to drug interactions in other systems including the most worrisome pathogenic microbes and cancer.

## Materials and Methods

### Strains, media, and drugs

Deletion strains are from the Keio collection of 3,985 nonessential gene deletions (Baba *et al*, 2006). Since the strains in this collection have a kanamycin resistance marker, we introduced kanamycin resistance on a low-copy-number plasmid (pUA66; Zaslaver *et al*, 2006) into the parent strain (BW25113, ‘WT’). All gene deletion mutants with clear effects on drug interactions (*pgpA, gmhB, metL, ppiD, lpxM, atpF, rcsD, dnaK, atpC, rfaG, rfaP, rfaC, lpcA, rep, spr*) were verified by sequencing; the correct gene deletion was confirmed in all cases. All experiments were performed in lysogeny broth (LB) medium. Drugs were obtained from Sigma-Aldrich (catalogue numbers: ciprofloxacin, 17850; chloramphenicol, C0378; mecillinam, 33447; nitrofurantoin, N7878; tetracycline, 268054; trimethoprim, 92131). Drug stocks were prepared in water (ciprofloxacin, mecillinam), ethanol (chloramphenicol, tetracycline, trimethoprim) or dimethylformamide (nitrofurantoin), passed through a 0.22-μm filter, and stored in the dark at −20°C. Drugs were used at fixed concentrations that inhibit wild-type growth by ∼30% (Table1); growth remained exponential at the concentrations used (Fig1D and E). In drug combination experiments, the same concentration as in the single drug experiments was used except for mecillinam–trimethoprim for which no growth occurred at these concentrations due to its synergistic interaction; therefore, the concentration of both drugs was reduced in the combination experiment (20 ng/ml for mecillinam and 50 ng/ml for trimethoprim). The resulting growth inhibition of the wild-type was ∼60% for chloramphenicol–tetracycline, ∼50% for chloramphenicol–trimethoprim, ∼20% for ciprofloxacin–tetracycline, ∼55% for mecillinam–trimethoprim, ∼35% for trimethoprim–tetracycline, and ∼30% for nitrofurantoin–chloramphenicol. Thiamin pyrophosphate (Sigma-Aldrich catalogue number C8754) was dissolved in water and stored in the dark at −20°C. Oligomycin A and venturicidin A (Szabo Scandic catalogue numbers SACSC-201551A and SACSC-202380A) were dissolved in ethanol and stored in the dark at −20°C.

### Growth rate measurements

Each strain was incubated for ∼20 h in one well of a 96-well plate (nontreated transparent flat bottom, Nunc) containing 200 μl medium. Cultures were inoculated using a replicator (V&P Scientific) transferring ∼0.2 μl from a (thawed) overnight culture kept at −80°C with 15% glycerol. Optical density (OD) at 600 nm was measured every ∼30 min in a plate reader (Tecan Infinite F500, 5 flashes, 10 ms settle time; filter: D600/20×; Chroma). The plates were incubated in an automated incubator (Liconic Storex) kept at 30°C, > 95% humidity, and shaken at 720 rpm. In addition, directly before each measurement, plates were shaken on a magnetic shaker (Teleshake; Thermo Scientific) at 900 rpm for 20 s. A customized liquid handling robot (Tecan Freedom Evo 150) was used to automate these experiments and measure over 2,000 growth curves per day. To achieve nearly identical growth conditions for all strains in each condition, the growth curves of all ∼4,000 deletion strains were measured over two consecutive days using the same freshly prepared drug solution. The growth rate in exponential phase was quantified from the OD increase over time by a linear fit of log(OD) in the range 0.022 < OD < 0.22 (magenta lines in Fig1). Late growth occurring after 1,000 min was discarded because in rare cases, fast growing strains (likely resistant mutants) overtook the population. For mecillinam, only early growth (happening before 450 min) was considered because many instances of late fast growth occurred for this drug; this effect may be due to drug decay as mecillinam is relatively unstable (Baltzer, 1979). All growth rates were normalized to the growth rate of the parent strain in the absence of drug measured on the same day. These automated measurements led to highly reproducible growth rates: replicate measurements of the entire deletion collection under chloramphenicol on different days showed a Pearson correlation of 0.94 (Supplementary Fig S1A); replicates of growth rate measurements had a variation coefficient (standard deviation over mean) of typically < 5%. Media evaporation from plates and edge effects were virtually undetectable. Mutant sensitivities to antibiotics determined from these data were consistent with published data (Supplementary Fig S10).

### Two-drug response surfaces

For each drug pair, response surfaces were measured for wild-type and 18 gene deletion mutants, covering outlier mutants with strong differences between observed and expected growth rate (Fig3A and B; Supplementary Fig S5) and additional mutants that showed clear changes in sensitivity to at least one of the constituent drugs. Response surfaces were measured using 12 × 8 or 24 × 24 two-dimensional drug concentration matrices set up with a liquid handling robot across one (12 × 8) or six 96-well plates (24 × 24), respectively. The concentration profile for each drug was set up according to 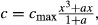 where *c*_max_ was the highest concentration used, *x* was linearly spaced from 0 to 1 with 8, 12, or 24 steps depending on the experiment, and *a* = 1/3. This concentration profile was chosen to adequately sample the relevant part of the two-drug response surface where growth rate changes significantly. The points in two-dimensional concentration space where growth was measured are shown by small gray dots in all Figures. For the representation of two-dimensional response surfaces, we used the optical density 12 h after inoculation instead of the growth rate because this quantity was slightly more reproducible and yielded smoother response surfaces; this representation does not affect any of the conclusions on drug interaction changes (Supplementary Fig S1B–D). Smooth surfaces and isoboles (contour lines) were calculated by linear interpolation (Matlab function *interp2*) of the experimental data. The IC_50_ line is the isobole of 50% growth inhibition, and in practice, we used the isobole of 90% growth inhibition as the MIC (Minimal Inhibitory Concentration) line. We measured the response surface of the wild-type and of all mutants that showed a clear change in drug interaction (Fig3C; Supplementary Fig S7) at least in duplicate; replicate response surfaces measured on different days were generally highly reproducible (Supplementary Fig S1B–D) and, in particular, all drug interaction changes in mutants were confirmed.

### Expected growth rate in drug combinations

We calculated the expected growth rate (Fig2D; Supplementary Fig S5) for each mutant strain *i* in the combination of drugs A and B at concentration *a* and *b*, respectively, using the following procedure:

- The effective concentration 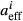 of drug A experienced by mutant *i* was calculated from the response 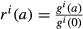 of this mutant to drug A at concentration *a* alone; here, *g^i^*(*a*) and *g^i^*(0) denote the growth rate of mutant *i* in the presence and in the absence of drug A, respectively. Specifically, this was done by identifying the concentration in the WT dose–response curve *r*^WT^(*a*), which corresponds to the same response; that is we determined 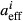 such that 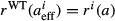; analogously, we calculated 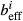. The scaling factors for the drug concentration are then given by 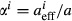 and 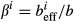 and that for the growth rate is given by *γ*^*i*^ = *g*^*i*^(0)/*g*^WT^(0). This procedure exploits the observation that the dose–response curve *r*^i^ (*a*) of mutants is generally the same as the wild-type curve with a linearly rescaled drug concentration.
- The interaction coefficient *I*^WT^ was calculated at position 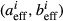 in the two-drug space (Fig2C). This interaction coefficient of the WT was defined as the measured response compared to the Bliss additive expectation 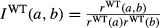 (Yeh *et al*, 2006). The WT response surfaces for all drug pairs were measured in a fine resolution 24 × 24 concentration matrix to enable the precise determination of *I*^WT^.
- The expected growth rate of mutant *i* in the combination of both drugs at concentrations *a*, *b* was then 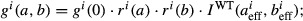 this equation formalizes the assumption that the interaction coefficient is a universal invariant and, for all mutants, is the same as in the WT at the effective drug concentrations.

This procedure yielded accurate predictions of growth rate under drug combinations (Fig2D; Supplementary Fig S5). For mecillinam–trimethoprim, we had to slightly adjust this procedure since the concentrations used in the drug combination had to be reduced (see ‘Strains, media, and drugs’ above). We took this into account by multiplying 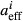 and 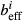 with a constant factor capturing the reduced drug concentrations. Mutants that deviated from this expectation were used to identify altered drug interactions (Fig3, Supplementary Fig S5); some mutants deviated from this expectation for other reasons, in most cases because they had extremely long lag phase. To estimate the error of the expected growth rate, we added a 5% normal distributed relative error (empirically determined, see ‘Growth rate measurements’ above), and an estimated absolute error of 0.01 (capturing the limited reproducibility of extremely low growth rates) to each growth rate measurement. The standard deviation at each expected growth rate was then numerically calculated from 10,000 randomly sampled growth rates *g*(*a*) and *g*(*b*) (from a uniform distribution between 0 and 1) for each drug pair. The dashed lines in Figs2D and 3A and B, and Supplementary Fig S5 show two standard deviations (corresponding to a 95% confidence interval) for both the expected (*x*-axis) and the measured growth rate in the combination (*y*-axis). Due to differences in response surface shape, the resultant error estimates depend strongly on the drugs used. The density scatterplots in these and other Figures were generated using the scatplot function available at http://www.mathworks.com/matlabcentral/fileexchange/8577-scatplot.

### Gene ontology enrichment analysis

To identify mutants whose growth rate in the drug combination deviated strongly from the expectation (outliers in Fig3A and B; Supplementary Fig S5), we performed an orthogonal regression using principal component analysis (Matlab function *princomp*) and used the orthogonal distance to the regression line to quantify deviations from the expectation. We performed gene ontology enrichment analysis (Fig3D; Supplementary Table S1) on the 30 outliers with the strongest deviation from the expectation. We excluded outliers for which both the expected and the observed growth rates were below 0.15 because these extremely low growth rates are hard to quantify reliably. The gene ontology database used in our analysis was retrieved from geneontology.org (released 07/15/2014) and the gene association file linking gene names to GO numbers from ecocyc.org (GOC validation date: 06/26/2014) (Keseler *et al*, 2005). The *P*-values were obtained using a custom implementation of Sherlock and Weng's GO:Termfinder software (Tavazoie *et al*, 1999) and Bonferroni corrected for the number of GO terms tested.

### Gene expression measurements

Transcriptional regulation of the *atpI* promoter was measured as a proxy for ATP synthase expression in concentration gradients of different antibiotics using a promoter–GFP reporter strain (Zaslaver *et al*, 2006) and quantified as described (Bollenbach *et al*, 2009).

## Supporting Information

Supplementary Table S1

Review Process File
