# Supplementary material for: Systematic discovery of drug interaction mechanisms
Source: Mol Syst Biol. 2015 Apr 29;11(4):807. doi: 10.15252/msb.20156098 (PMC4422561; doi:10.15252/msb.20156098)
Supplement: Supplementary file 1 [file msb0011-0807-sd1.pdf]

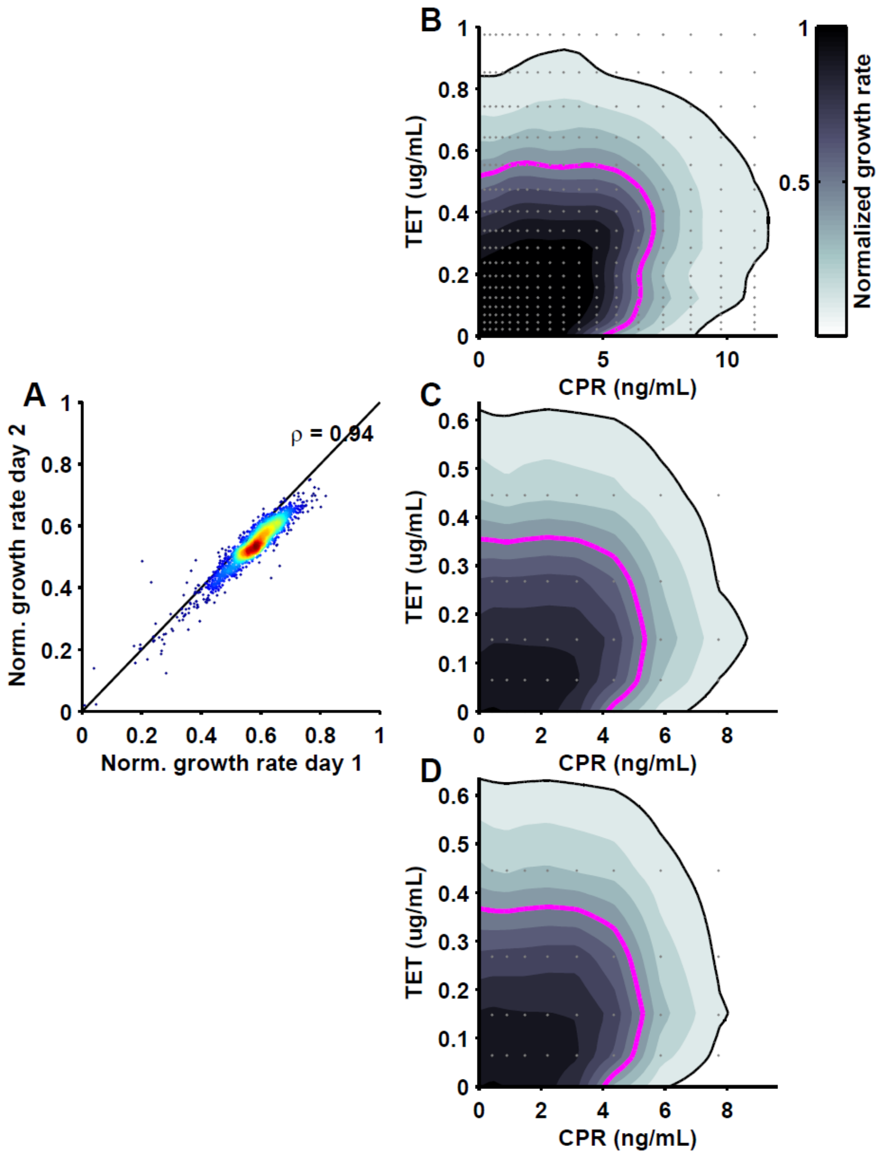

Figure S1. Reproducibility of growth measurements. (A) Density scatterplot of two replicate measurements of growth rates of genome-wide gene deletion strains (Baba et al., 2006) in chloramphenicol (1µg/mL), made six months apart. The data points are slightly but systematically off the diagonal because of a small difference in drug concentration between the replicates. The Pearson correlation coefficient is 0.94. (B-D) Response surface of WT in two-dimensional concentration gradient of ciprofloxacin and tetracycline measured in replicates on three different days and showing growth rate (B) or optical density 12h after inoculation (C,D); Materials and Methods. While there are inevitable small shifts in the MIC values between replicates (due to the difficulty of preparing exactly the same drug condition on different days) the shape of the response surfaces is highly reproducible.
