# Supplementary material for: Systematic discovery of drug interaction mechanisms
Source: Mol Syst Biol. 2015 Apr 29;11(4):807. doi: 10.15252/msb.20156098 (PMC4422561; doi:10.15252/msb.20156098)
Supplement: Supplementary file 2 [file msb0011-0807-sd2.pdf]

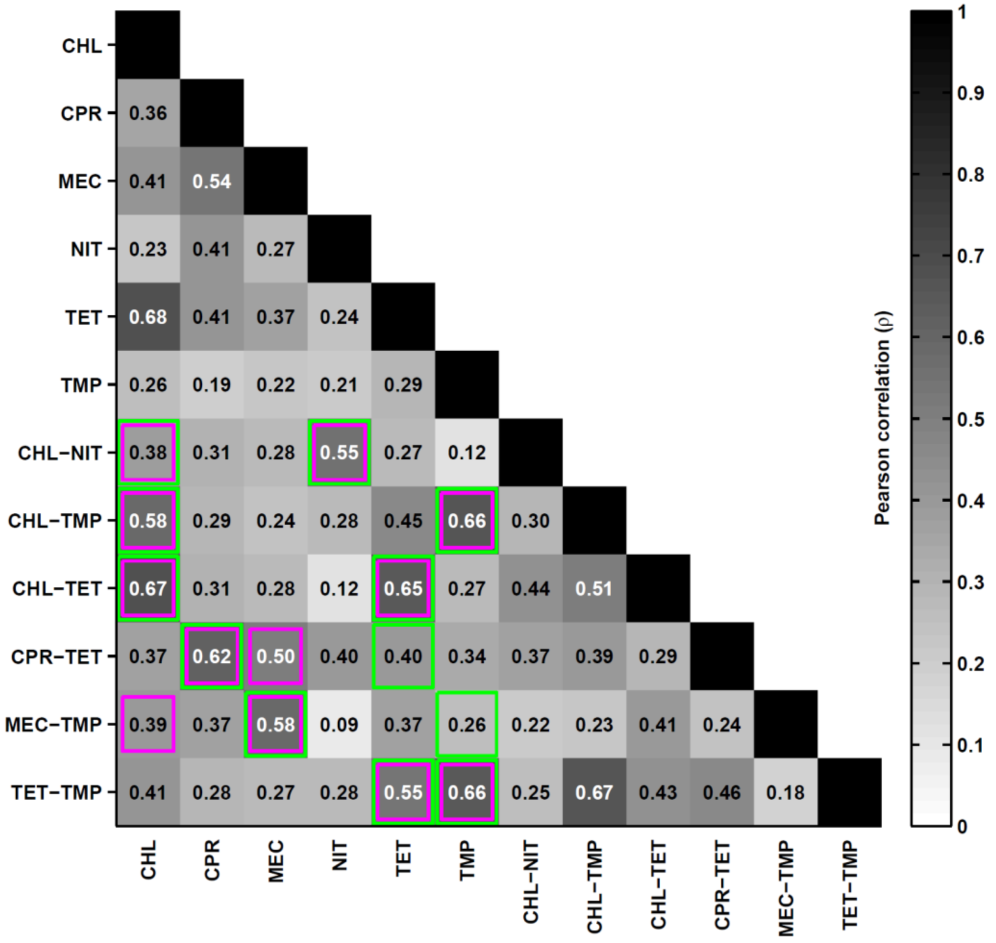

Figure S2. Comparison of the effects of different drugs and drug combinations on growth of genome-wide mutants. Matrix of Pearson correlation coefficients between growth responses of genome-wide gene deletion mutants to drugs and drug combinations used in this study (Table 1); the response of a mutant is defined as its growth rate in a drug condition normalized to its growth rate in the absence of drug (Materials and Methods). For each drug combination, the tiles showing the correlation with its two constituent drugs are highlighted by green frames and the two largest correlation coefficients with the individual drugs are highlighted by magenta frames. In most cases, the correlation with the constituent drugs is highest, consistent with the view that drug combination effects are essentially a superposition of the constituent drug effects. However, the combination of ciprofloxacin and tetracycline has higher similarity to mecillinam than to tetracycline; this effect is largely explainable by the fact that ciprofloxacin alone is surprisingly similar to mecillinam. The combination of mecillinam and trimethoprim is more similar to chloramphenicol, tetracycline, and ciprofloxacin than to trimethoprim, suggesting that this synergistic drug pair causes perturbations to cell physiology that are not a simple superposition of the constituent drug effects. Trimethoprim-tetracycline and trimethoprim-chloramphenicol combinations were more similar to each other than to their respective constituent drugs, likely reflecting the simultaneous inhibition of folic acid and protein synthesis in both of these combinations.
