# Supplementary material for: Systematic discovery of drug interaction mechanisms
Source: Mol Syst Biol. 2015 Apr 29;11(4):807. doi: 10.15252/msb.20156098 (PMC4422561; doi:10.15252/msb.20156098)
Supplement: Supplementary file 3 [file msb0011-0807-sd3.pdf]

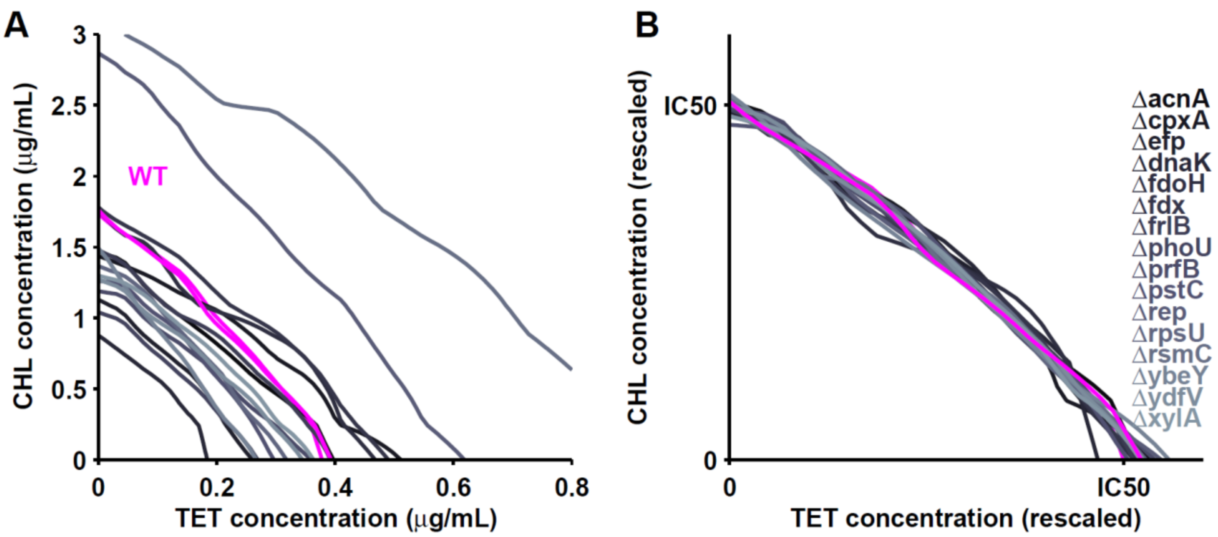

Figure S3. The additive drug interaction between chloramphenicol and tetracycline is robust to genetic perturbations. (A) Lines of 50% growth inhibition (IC_{50} lines) in two-dimensional concentration gradient of chloramphenicol and tetracycline for WT (magenta) and 16 mutants (gray) whose growth rate under the drug combination deviated most strongly from expectation (Fig. S5). (B) As A but with drug concentrations linearly rescaled to maximize similarity between mutants and WT. The drug interaction remains unchanged in all mutants, supporting that the additive chloramphenicol-tetracycline drug interaction is completely robust to genetic perturbations. Full response surfaces for three of these mutants are shown in Fig. S4.
