# Supplementary material for: Systematic discovery of drug interaction mechanisms
Source: Mol Syst Biol. 2015 Apr 29;11(4):807. doi: 10.15252/msb.20156098 (PMC4422561; doi:10.15252/msb.20156098)
Supplement: Supplementary file 4 [file msb0011-0807-sd4.pdf]

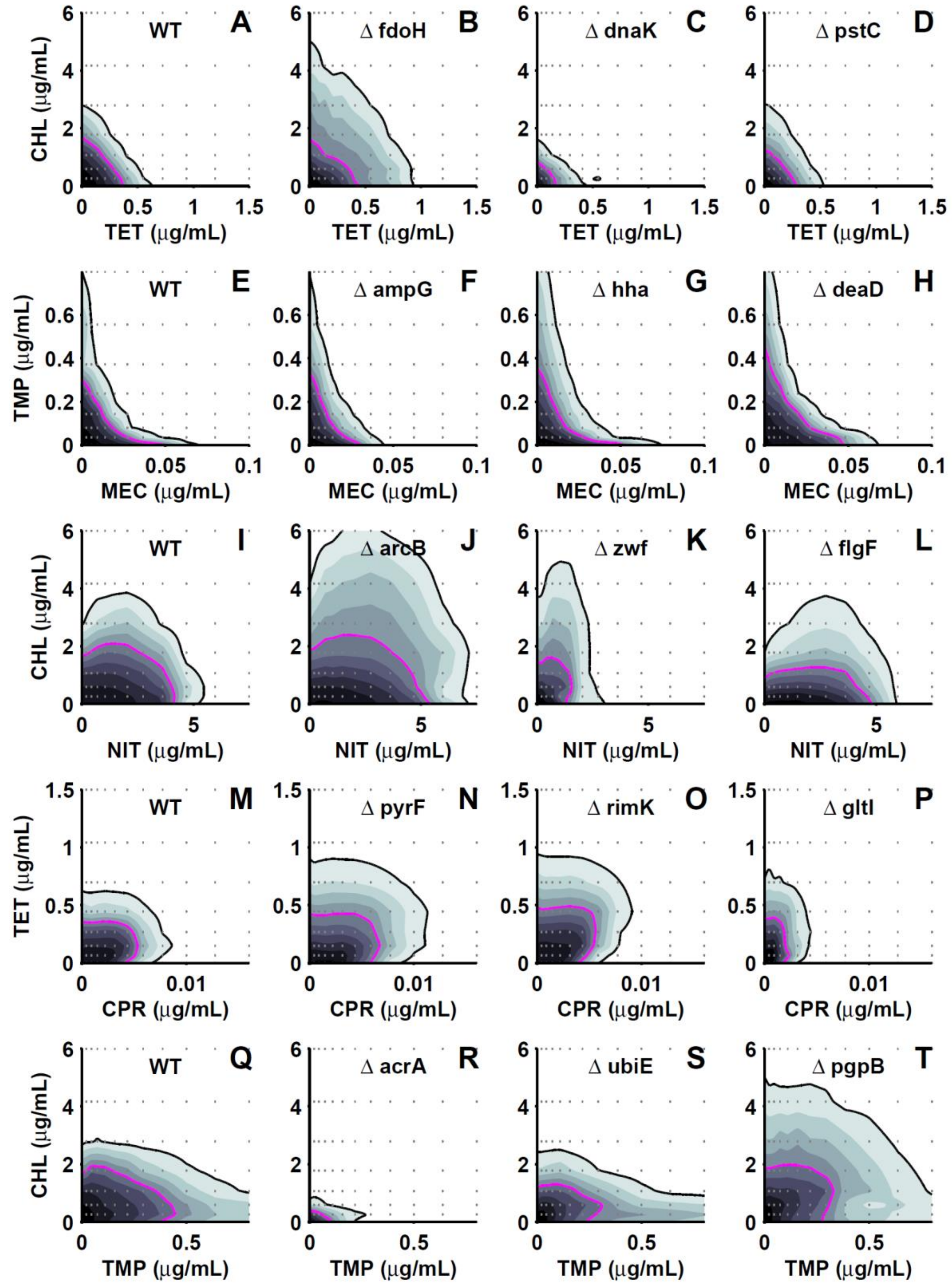

Figure S4. Additional examples of mutants that do not affect drug interactions. As Fig. 2A-F, for various mutants and drug pairs: (A-D) chloramphenicol-tetracycline, (E-H) trimethoprim-mecillinam, (I-L) chloramphenicol-nitrofurantoin, (M-P) tetracycline-ciprofloxacin, (Q-T) chloramphenicol-trimethoprim.
