# Supplementary material for: Systematic discovery of drug interaction mechanisms
Source: Mol Syst Biol. 2015 Apr 29;11(4):807. doi: 10.15252/msb.20156098 (PMC4422561; doi:10.15252/msb.20156098)
Supplement: Supplementary file 5 [file msb0011-0807-sd5.pdf]

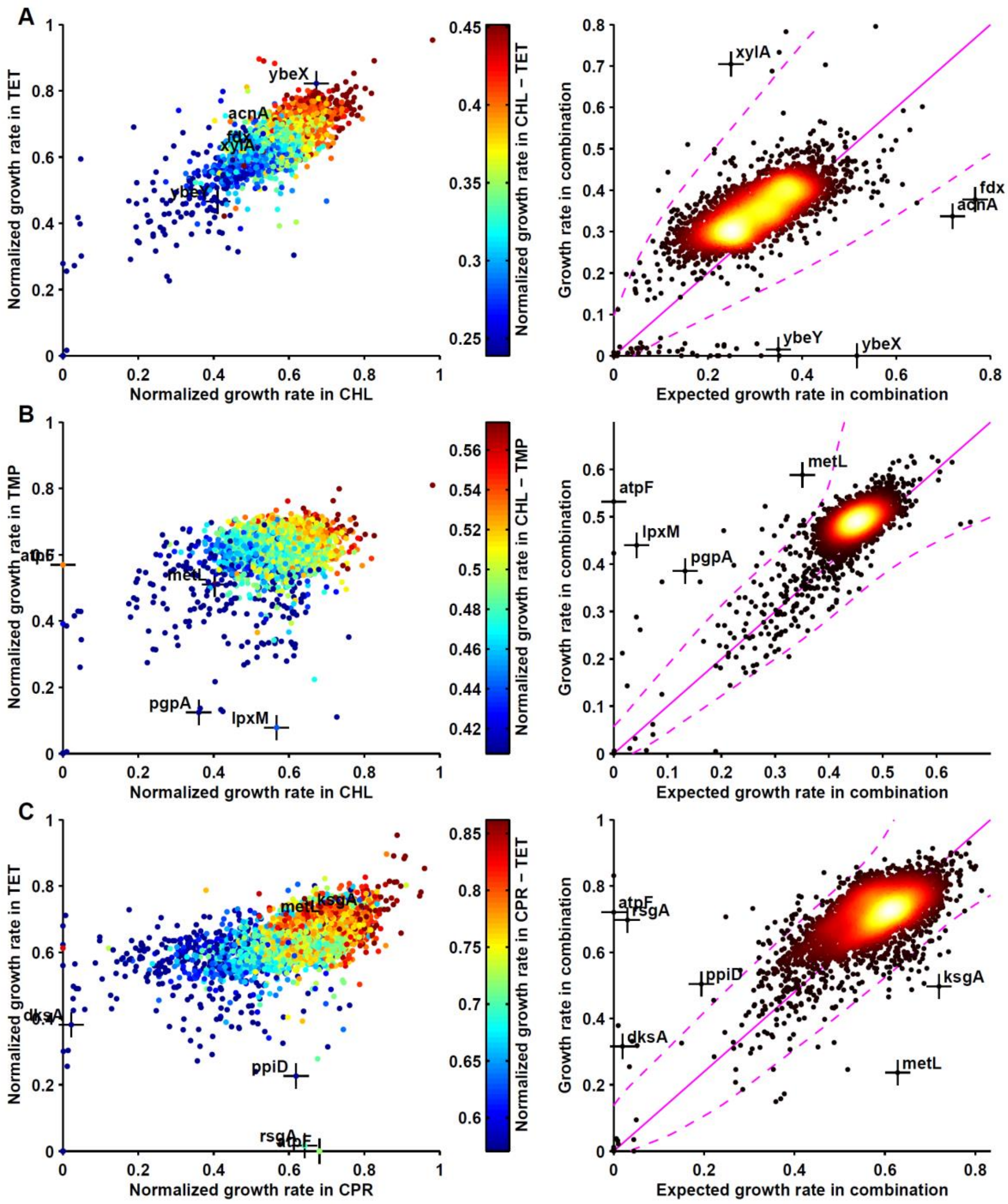

A
B
C
Normalized growth rate in TET
Normalized growth rate in CHL
Normalized growth rate in CHL – TET
Growth rate in combination
Expected growth rate in combination
Normalized growth rate in TMP
Normalized growth rate in CHL – TMP
Normalized growth rate in CPR
Normalized growth rate in CPR – TET
ybeX
ybeY
acnA
fdx
xylA
atpF
lpxM
pgpA
metL
ksgA
rsgA
ppiD
dksA

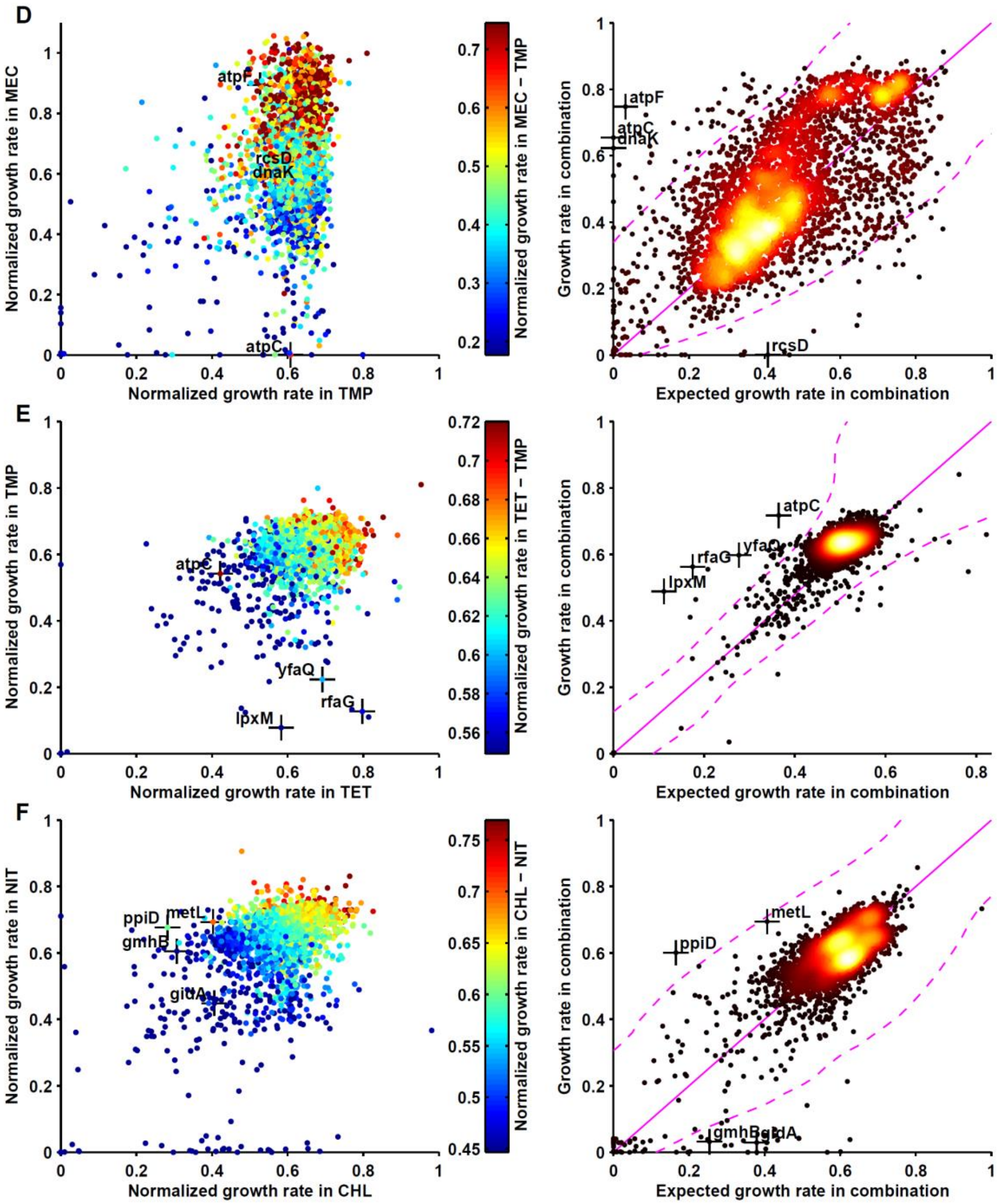

Figure S5. Growth rates of genome-wide deletion mutants under drug combinations and deviation from expected growth rates for all drug pairs. Scatterplots of measured growth rates of gene deletion mutants under drug combinations and constituent drugs as in Fig. 1F (left panels), and comparison of expected and observed growth rates as in Fig. 2D (right panels) for chloramphenicol-tetracycline (A), chloramphenicol-trimethoprim (B), ciprofloxacin-tetracycline (C), trimethoprim-mecillinam (D), tetracycline-trimethoprim (E), and chloramphenicol-nitrofurantoin (F). For ciprofloxacin-tetracycline and trimethoprim-tetracycline, the data points in the right panels are slightly but systematically off the diagonal because of small differences in drug concentration between the combination and individual drug measurements; these occurred because the individual and combined drug conditions had to be prepared on different days. To adjust the shown 95% confidence intervals for this effect the slope of the magenta lines was slightly increased for these two drug pairs.
