# Supplementary material for: Systematic discovery of drug interaction mechanisms
Source: Mol Syst Biol. 2015 Apr 29;11(4):807. doi: 10.15252/msb.20156098 (PMC4422561; doi:10.15252/msb.20156098)
Supplement: Supplementary file 6 [file msb0011-0807-sd6.pdf]

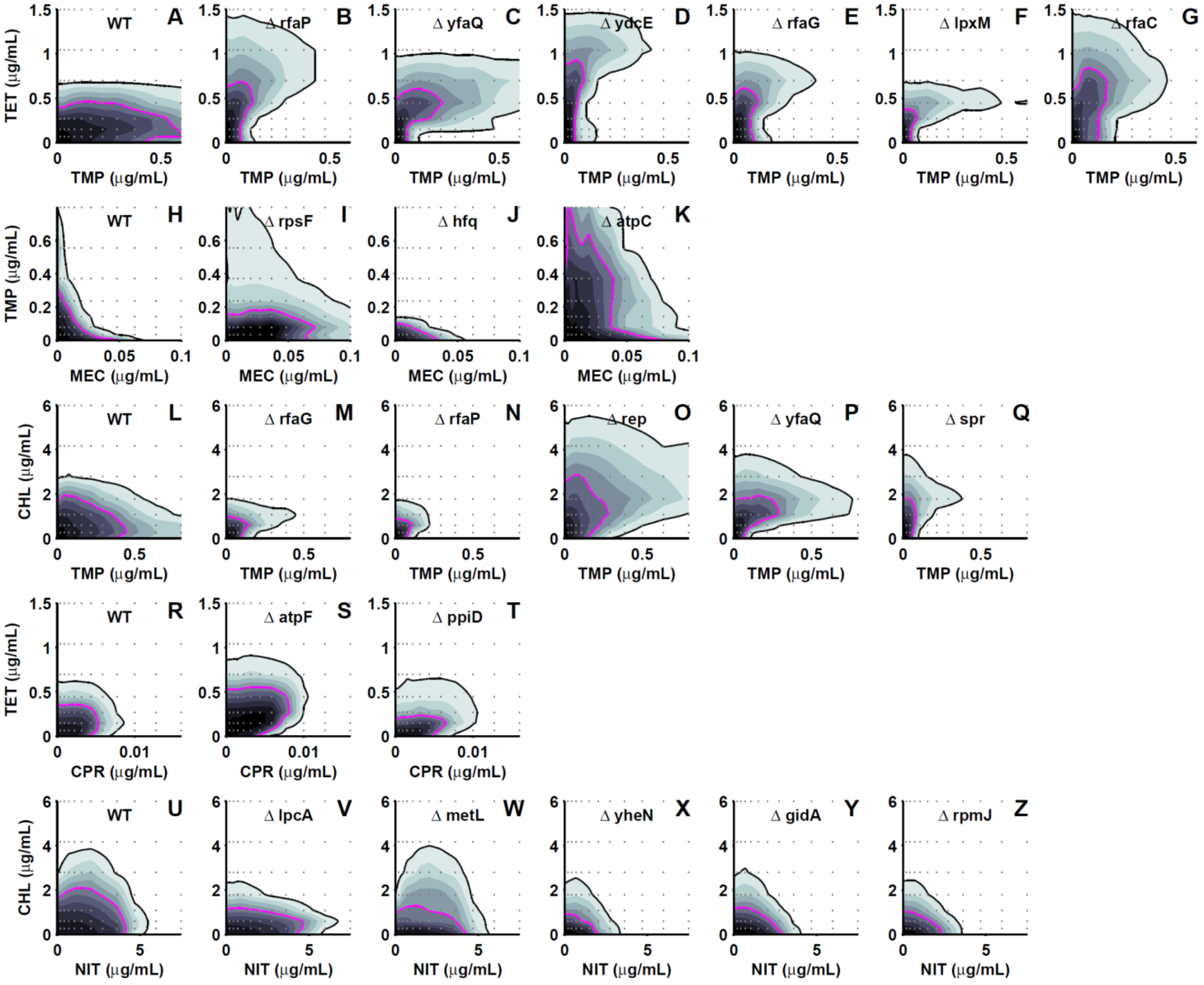

Figure S6. Additional mutants with altered drug interactions. As Fig. 3C, for additional mutants that change the drug interactions between different drug pairs: (A-G) tetracycline-trimethoprim, (H-K) trimethoprim-mecillinam, (L-Q) chloramphenicol-trimethoprim, (R-T) tetracycline-ciprofloxacin, (U-Z) chloramphenicol-nitrofurantoin.
