# Supplementary material for: Systematic discovery of drug interaction mechanisms
Source: Mol Syst Biol. 2015 Apr 29;11(4):807. doi: 10.15252/msb.20156098 (PMC4422561; doi:10.15252/msb.20156098)
Supplement: Supplementary file 7 [file msb0011-0807-sd7.pdf]

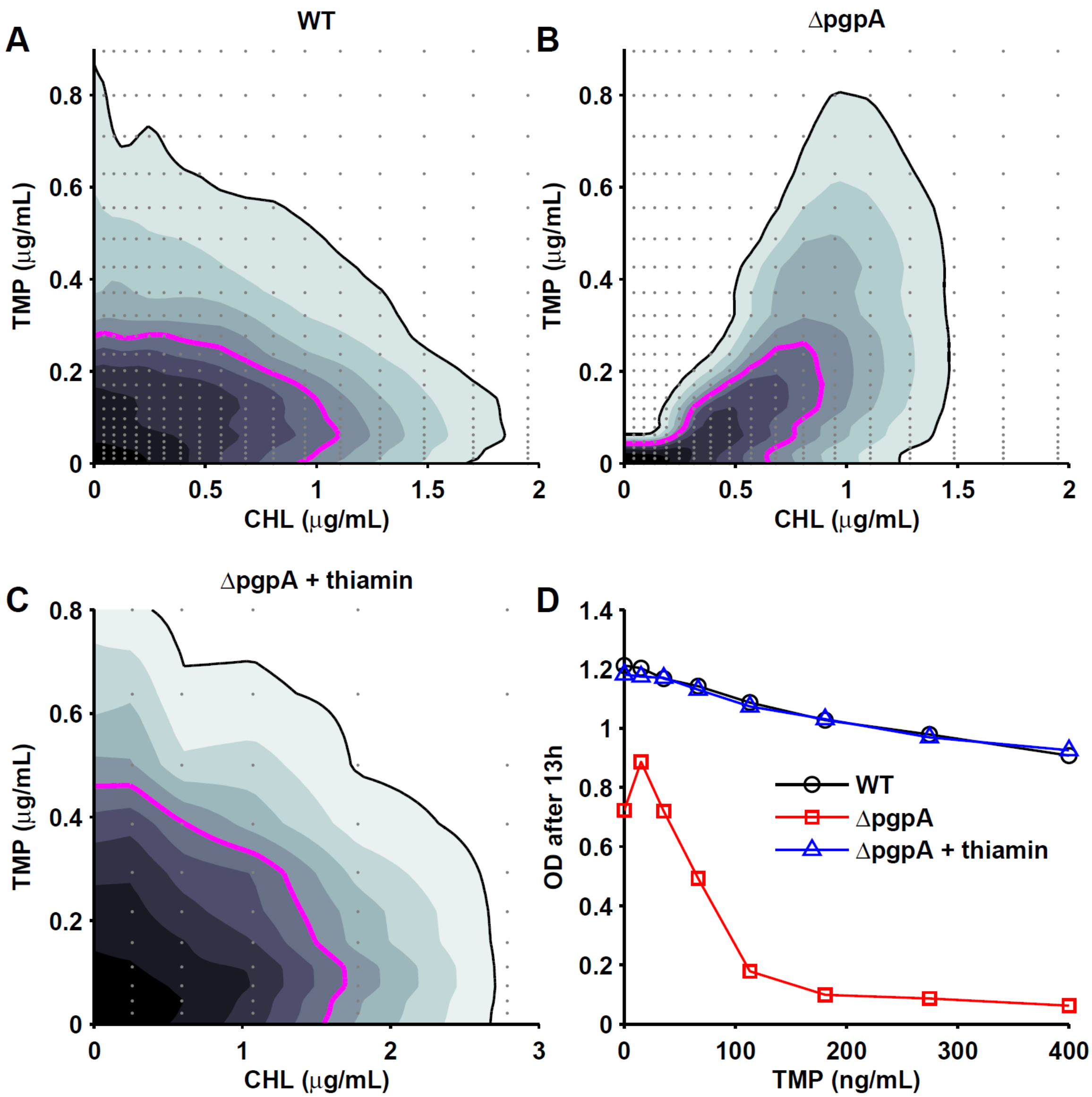

Figure S7. A thiamin synthesis hypomorph mutant leads to reciprocal suppression. Growth of WT (A) and *pgpA* deletion mutant (B) in two-dimensional chloramphenicol-trimethoprim concentration gradient (Materials and Methods). The antagonistic interaction in the WT becomes reciprocally suppressive in the *pgpA* mutant; at the same time, the *pgpA* mutant has greatly increased sensitivity to trimethoprim. This effect is due to truncation of the neighboring thiamin synthesis gene *thiL* which is essential, has overlapping open reading frame with *pgpA* (Keseler *et al*, 2005) and thus likely becomes hypomorphic as a result of *pgpA* deletion. (C) As B but supplemented with thiamin pyrophosphate at 80ng/mL (Materials and Methods): reciprocal suppression in *pgpA* mutant reverts to an antagonistic drug interaction similar to WT (A). Experiment was done on a different day and with different concentration sampling than those in A,B, leading to slight differences in MICs. (D) Trimethoprim dose-response curves (OD after 13h) of WT and *pgpA* mutant with and without supplementation of thiamin as in C; trimethoprim sensitivity of the *pgpA* mutant is completely rescued by thiamin supplementation.
