# Supplementary material for: Systematic discovery of drug interaction mechanisms
Source: Mol Syst Biol. 2015 Apr 29;11(4):807. doi: 10.15252/msb.20156098 (PMC4422561; doi:10.15252/msb.20156098)
Supplement: Supplementary file 8 [file msb0011-0807-sd8.pdf]

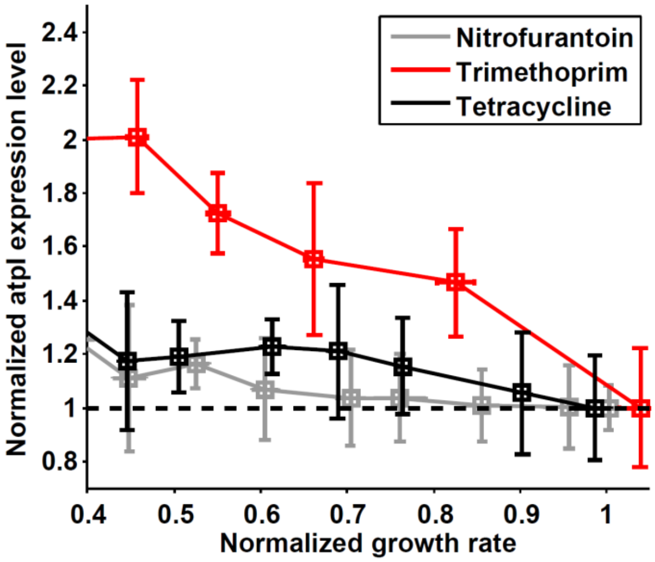

Figure S8. ATP synthase expression increases in response to trimethoprim. Transcriptional regulation of the *atpI* promoter (which controls expression of ATP synthase genes) in response to trimethoprim (red), nitrofurantoin (gray), and tetracycline (black) as a function of growth rate at different drug concentrations (Materials and Methods). Transcription from the *atpI* promoter increased two-fold in response to trimethoprim at ~60% growth inhibition; in contrast, no significant increase occurred for tetracycline and nitrofurantoin which were used as reference.
