# Supplementary material for: Systematic discovery of drug interaction mechanisms
Source: Mol Syst Biol. 2015 Apr 29;11(4):807. doi: 10.15252/msb.20156098 (PMC4422561; doi:10.15252/msb.20156098)
Supplement: Supplementary file 9 [file msb0011-0807-sd9.pdf]

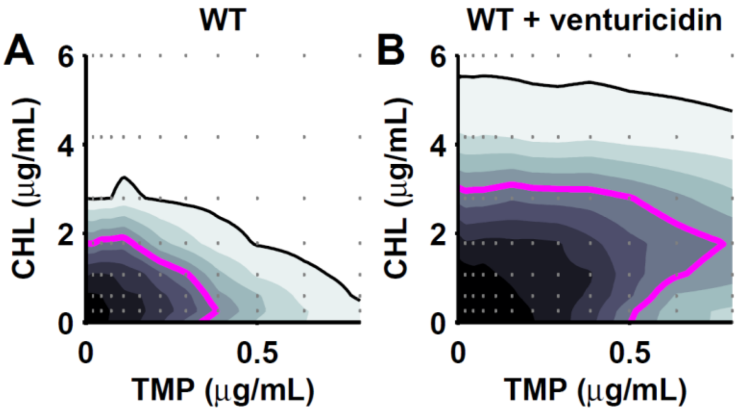

Figure S9. ATP synthase inhibition by venturicidin leads to synthetic suppression. Growth of WT in two-dimensional chloramphenicol-trimethoprim concentration gradient in the absence (A) and in the presence (B) of the ATP synthase inhibitor venturicidin at 32μg/mL. Note change of drug interaction from antagonistic to suppressive, validating the effect of oligomycin, a different ATP synthase inhibitor (*cf.* Fig. 4C,D). For unknown reasons, venturicidin increases chloramphenicol MIC by a factor of 2, similar to oligomycin, indicating a suppressive interaction between these drugs (*cf.* Fig. 4C,D). There is no clear indication for any drug interaction between trimethoprim and venturicidin or oligomycin.
