# Supplementary material for: Systematic discovery of drug interaction mechanisms
Source: Mol Syst Biol. 2015 Apr 29;11(4):807. doi: 10.15252/msb.20156098 (PMC4422561; doi:10.15252/msb.20156098)
Supplement: Supplementary file 10 [file msb0011-0807-sd10.pdf]

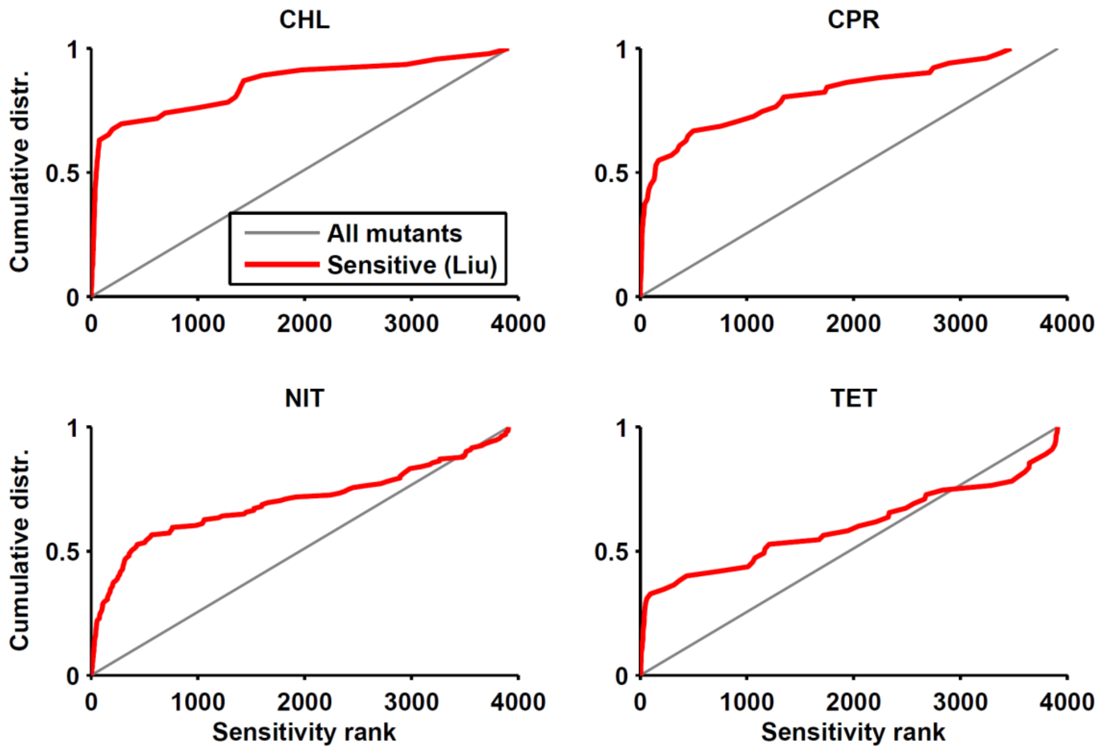

Figure S10. Sensitivity of mutants to antibiotics determined from growth rate measurements is consistent with previous measurements of MIC changes. Red lines show cumulative distributions of the sensitivity rank of gene deletion strains that were reported to be sensitive to chloramphenicol, ciprofloxacin, nitrofurantoin, and tetracycline, respectively, based on MIC measurements (Liu *et al*, 2010); the sensitivity rank is the rank of the mutant when all deletion mutants are sorted in order of decreasing sensitivity based on their growth response to each drug in our data set (i.e. low rank corresponds to high sensitivity). The majority of strains identified as sensitive in (Liu *et al*, 2010) are also among the most sensitive ones in our data set.
