# Supplementary material for: Systematic discovery of drug interaction mechanisms
Source: Mol Syst Biol. 2015 Apr 29;11(4):807. doi: 10.15252/msb.20156098 (PMC4422561; doi:10.15252/msb.20156098)
Supplement: Supplementary file 12 [file msb0011-0807-sd12.pdf]

molecular systems biology

Systematic discovery of drug interaction mechanisms

Guillaume Chevereau and Tobias Bollenbach

Corresponding author: Tobias Bollenbach, IST Austria

Review timeline:

Submission date:	12 February 2015
Editorial Decision:	20 March 2015
Revision received:	09 April 2015
Accepted:	15 April 2015

Editor: Maria Polychronidou

Transaction Report:

(Note: With the exception of the correction of typographical or spelling errors that could be a source of ambiguity, letters and reports are not edited. The original formatting of letters and referee reports may not be reflected in this compilation.)

1st Editorial Decision 20 March 2015

Thank you again for submitting your work to Molecular Systems Biology. We have now heard back from the three referees who agreed to evaluate your manuscript. As you will see below, the referees think that the presented findings are interesting. They list however a series of concerns, which we would ask you to address in a revision of this work. Most of the reviewers' comments refer to the need to include further discussion on several points and are quite clear, so there is no need to repeat them. Regarding the comment of referee #3 on changing the title, we think that it is not necessary and therefore we do not object to keeping the current title.

Reviewer #1:

The authors use a non-essential gene knockout collection in E coli to identify genetic mechanisms underlying the growth response to pair-wise antibiotic challenge. One of the most exciting contributions of this work is the methodology they introduce that allows inevitable perturbations to growth physiology to be disentangled from specific mechanisms underlying the response: the majority of mutants exhibit a modified growth-rate response to the singlet antibiotics which can be compensated by a simple rescaling of the antibiotic concentration, and this same rescaling persists in the pair-wise growth-response surface. What is remarkable is that those mutants with deviant growth-response immediately identify themselves as candidate mediators of the specific drug interaction. In this way, candidate genes are narrowed down from thousands to less than a dozen. As proof-of-principle, mutants defective in polysaccharide and ATP synthesis are identified as primary mediators of dramatic changes to drug interactions for several antibiotic pairs.

In all, the study is well-done. I have only minor suggestions regarding presentation. The method of response rescaling in the singlet case to predict rescaling in the pair-wise case is of general applicability beyond drug-interactions. The same principle could be applied to any orthogonal pairs

of challenges (osmotic stress, temperature, pH, etc.) to identify a short-list of genetic candidates mediating paired-response. The rescaling principle is currently briefly discussed at the bottom of page 2, in the caption for Fig 2 and then again in the 'Materials and Methods.' Because it is the foundation of the study, the authors may consider devoting a paragraph to explaining the principle in detail, or perhaps even a Box that contains panels from Fig 2 and a synopsis of the 'Expected growth rate in drug combinations' section in the 'Methods.'

Furthermore, the notation is difficult to parse between the main text and the methods - lambda is used in the main text, but does not appear in the methods for example. Nor is it clear that the simple expression for the mutant growth surface shown in the main text is the same as the more complicated expression in part (iii) of the 'expected growth' section in the methods (this point goes beyond a unity of notation). Instead of lambda, if 'a' and 'b' are used to denote the concentration, 'alpha' and 'beta' may be a more transparent choice for the rescaling parameters.

Reviewer #2:

The paper by Chevereau and Bollenbach describes a systematic characterization of how genetic perturbations (genome wide knock outs) affect drug interaction type. Using the KEIO collection and a high throughput microplate based growth profiling setup they characterize the growth rate increase or decrease in the presence of 6 different drug combinations and their component drugs. They find that relatively few knock outs affect drug interactions and notably these knock outs cluster in a few cellular functions. The authors hypothesize about the possibly mechanisms and with a clever use of specific inhibitors and strains, they provide significant support for their hypothesis. I believe that this is excellent and timely work and it should be published with minimal delay. It will have a significant impact on the thinking in the field of antibiotic combination therapy.

I have a few minor points that could be addressed through slight revisions in the text:

(1) In the introduction when describing the how genetic perturbations affect drug interactions the authors should consider also the recent work from the Sommer lab (Munck et.al. 2014, Evgrafov et.al. 2015) where the authors show that drug interactions are modulated by resistance evolution highlighting the evolutionarily selected genetic perturbations tend to affect drug interactions. If could be interesting to study the mutations found in those studies to see if they support some of the conclusions of this paper (e.g. is there any overlap between the genes mutated in the earlier studies and the knock outs found to perturb drug interactions in this study).

(2) Also, the work of the Miller lab should be acknowledged (Liu et.al. 2010) in the discussion of the initial characterization of the KEIO collection. It would also be nice to cross validate the findings of Liu et.al. with the results of this study in the supplement. E.g. look at the overlap between the changes in susceptibility found previously for the KEIO collection with the changes in growth rate found in the present study.

(3) It appears from figure 2D that the tendency is towards antagonism for the genetic perturbation outliers. In Figure S4, there appears to be clear trends in either direction for each combination (also towads synergism in Fig S5 F). It would be beneficial to discuss this in the manuscript and possibly its relation to the WT interactions. The authors have already some discussion, but I would suggest that this is expanded.

Reviewer #3:

General Summary

The manuscript "Systematic discovery of drug interaction mechanisms" by Chevereau and Bollenbach is exciting primarily because of the new finding that the detailed 'shape' of drug interactions is generally preserved across diverse genetic perturbations, even where those perturbations substantially alter sensitivity of the organism to one drug or another. This is exciting because a single detailed measurement of drug interactions in one genetic background can then be

'warped' to predict the effects in other genetic backgrounds. Exceptions to this newly established phenomenon are therefore also interesting, and some of these are described and explored. The experiments seem generally well designed, and it was an impressive amount of work to phenotype individual strains as opposed to competitively-grown barcoded populations.

The follow-up studies are useful, and acceptably round out the contribution of the large-scale studies. However the two most fleshed out examples do not really constitute "systematic discovery of drug interaction mechanisms" that would support the manuscript's title. However, with a title that better captured the main contribution (highly predictive modeling of drug x drug x gene interactions from drug x drug interactions), publication of this study would represent a very useful contribution to the field.

There were some issues with clarity about how the drug/drug/gene trios were chosen for the detailed measurements of drug interaction. I'll summarize what I think was done overall and refer back to this below:

-Expt 1: 6 drug x genome experiments
-Expt 2: (drug A + drug B) x genome for 6 out of the 15 possible pairwise drug combinations
-Expt 3: 108 drug x drug experiments in particular gene mutant backgrounds
-Various followup experiments

Major Issues

-It was not at all clear how the 108 drug x drug x gene experiments were chosen. Was this based on Expt 2? If so how? Any biases in the selection process of 108 experiments could affect the downstream conclusions.

-"...the ATP synthase mutant atpF was more sensitive to trimethoprim and this sensitivity was reduced by chloramphenicol or mecillinam, leading to suppression (Fig. 3C); a similar effect occurred for ciprofloxacin-tetracycline (Fig. S6S). As DNA repair and synthesis require ATP (Waldstein et al., 1974), these observations suggest that the repair of DNA damage (caused by trimethoprim or ciprofloxacin) is impaired by low intracellular ATP concentration in the mutant; inhibiting protein synthesis should alleviate this impairment and increase growth as it reduces ATP turnover and increases intracellular ATP concentration (Schneider et al., 2002)."

The connection of ATP synthase with DNA repair is a reasonable speculation, but a great many cellular processes require ATP, and some subset of these (e.g., nucleotide biosynthesis, replication) will impact DNA repair indirectly. The observation that a phenotype depends on ATP does not point any more to DNA repair than it does to any other ATP-requiring process, so it cannot be claimed from this evidence that we now understand the mechanistic effects of atpF mutation on drug interaction. Other lines of evidence pointing to ATP level effects as the cause of changing drug interaction do not resolve this issue.

Moreover, even if we believe that lowering ATP levels inhibits DNA repair, it is not clear why oligomycin would change the nature of the chloramphenicol-trimethoprim interaction from antagonistic to suppressive, so can we say that we understand the mechanism of drug interaction.

Whether or not the interaction mechanisms are understood does not detract from what I saw as the main message of the paper, but if the authors want to claim they are revealing mechanisms, they should provide better arguments for this.

Minor Issues

-For Expt 2, the authors used a single mix of drug A and drug B at the same concentrations used in the single-drug experiments. The main text will be interpreted by most to mean that they diluted the drug combo to get 30% inhibition, but then the Materials and Methods says that they only diluted the drug combo for one pair that was dead, and otherwise used the same concentrations used for the single agents. Thus, the % inhibition must be generally higher than 30% for the drug combo experiments, but this is not discussed and % inhibitions are not provided.

-More importantly, Expt 2 seems irrelevant to the paper... I did not see any place where it was used outside of Fig 1F, and it was not clear what was learned from it there. If it wasn't used, this is not a major issue, and certainly the data should be reported either way, but if the authors intended to draw any conclusions from this experiment it should be made more clear. (Was it used to choose the 108 tests in Expt 3? See above.) At the very least the authors should tell us whether Expt 3 results agreed with Expt 2 results.

-Were there any replicates amongst the 108 drug x drug x gene experiments? Some sense of reproducibility of these studies should be provided?

-"Synergism and antagonism occur frequently between antimicrobials and are largely determined by the primary cellular target of the drugs that are combined (Cokol et al., 2011; Ocampo et al., 2014; Yeh et al., 2006)" While Ocampo et al and Yeh et al support this statement, the results of Cokol et al do not. In Cokol et al, most synergistic interactions amongst pairs of drugs with 'known targets' did not correspond to target pairs encoded by genes with negative genetic interactions. Drug interaction may be largely determined by the primary cellular target; however, results of Cokol et al suggest that for this to be true, drugs must generally target more and/or different gene products than those that are currently known.

-" Further, ATP synthase expression increased two-fold in response to trimethoprim (Fig. S8), suggesting that cells counteract ATP deficiency" Maybe "respond homeostatically to" would be more accurate than "counteract" as no evidence is presented that the deficiency has actually been remediated by increased ATP synthase expression.

-"Indeed, inhibiting ATP synthase led to suppression between trimethoprim and chloramphenicol..." Were oligomycin and venturicidin synergistic with trimethoprim?

- It would have been interesting to measure EDTA interaction with nitrofurantoin

1st Revision - authors' response 09 April 2015

(Please see next page)

Point-by-point response

Reviewer #1:

The authors use a non-essential gene knockout collection in E coli to identify genetic mechanisms underlying the growth response to pair-wise antibiotic challenge. One of the most exciting contributions of this work is the methodology they introduce that allows inevitable perturbations to growth physiology to be disentangled from specific mechanisms underlying the response: the majority of mutants exhibit a modified growth-rate response to the singlet antibiotics which can be compensated by a simple rescaling of the antibiotic concentration, and this same rescaling persists in the pair-wise growth-response surface. What is remarkable is that those mutants with deviant growth-response immediately identify themselves as candidate mediators of the specific drug interaction. In this way, candidate genes are narrowed down from thousands to less than a dozen. As proof-of-principle, mutants defective in polysaccharide and ATP synthesis are identified as primary mediators of dramatic changes to drug interactions for several antibiotic pairs.

In all, the study is well-done. I have only minor suggestions regarding presentation. The method of response rescaling in the singlet case to predict rescaling in the pair-wise case is of general applicability beyond drug-interactions. The same principle could be applied to any orthogonal pairs of challenges (osmotic stress, temperature, pH, etc.) to identify a short-list of genetic candidates mediating paired-response.

We thank the reviewer for the careful evaluation of our manuscript, his/her enthusiasm, and for the constructive comments. We agree that the rescaling principle we identified and validated for antibiotics likely holds more generally. We have added a sentence to the discussion at the end of the main text (p. 8, paragraph 3) where we now mention this broader relevance of our results, but also make clear that this will need to be validated in future work.

The rescaling principle is currently briefly discussed at the bottom of page 2, in the caption for Fig 2 and then again in the 'Materials and Methods.' Because it is the foundation of the study, the authors may consider devoting a paragraph to explaining the principle in detail, or perhaps even a Box that contains panels from Fig 2 and a synopsis of the 'Expected growth rate in drug combinations' section in the 'Methods.'

We agree that the rescaling principle is a central result of our study and that it makes our manuscript more accessible to explain this principle in more detail in the main text. To this end, we have split the part of the main text where this principle is introduced into two paragraphs (p. 5, paragraph 1-2); in the second paragraph we have added several sentences that explain essential details about this principle that were previously only in the Material and Methods. We left most formulae in the Material and Methods part because we felt that explaining all terms in these would disrupt the flow of the main text.

Furthermore, the notation is difficult to parse between the main text and the methods - lambda is used in the main text, but does not appear in the methods for example. Nor is it clear that the simple expression for the mutant growth surface shown in the main text is the same as the more complicated expression in part (iii) of the 'expected growth' section in the methods (this point goes beyond a unity of notation). Instead of lambda, if 'a' and 'b' are used to denote the concentration, 'alpha' and 'beta' may be a more transparent choice for the rescaling parameters.

We thank the reviewer for pointing out these difficulties with the notation. We have changed the symbols used for the drug concentration rescaling factors to α and β and to γ for the growth rate scaling factor (p. 5, paragraph 1). We further revised the Materials and Methods (section “Expected growth rate in drug combinations”) so that these rescaling factors are also explained there (p. 12,

paragraph 3). Regarding the expression for the mutant response surface, the simple expression in the main text is correct for the results shown in Fig. 2A,B which it refers to. However, it is true that the exact procedure for calculating the expected growth rate was previously only explained in the Materials and Methods section; we now explain the essence of this procedure in a new paragraph in the main text (p. 5, paragraph 2; see also the response to the previous point).

Reviewer #2:

The paper by Chevereau and Bollenbach describes a systematic characterization of how genetic perturbations (genome wide knock outs) affect drug interaction type. Using the KEIO collection and a high throughput microplate based growth profiling setup they characterize the growth rate increase or decrease in the presence of 6 different drug combinations and their component drugs. They find that relatively few knock outs affect drug interactions and notably these knock outs cluster in a few cellular functions. The authors hypothesize about the possibly mechanisms and with a clever use of specific inhibitors and strains, they provide significant support for their hypothesis. I believe that this is excellent and timely work and it should be published with minimal delay. It will have a significant impact on the thinking in the field of antibiotic combination therapy.

I have a few minor points that could be addressed through slight revisions in the text:

(1) In the introduction when describing the how genetic perturbations affect drug interactions the authors should consider also the recent work from the Sommer lab (Munck et.al. 2014, Evgrafov et.al. 2015) where the authors show that drug interactions are modulated by resistance evolution highlighting the evolutionarily selected genetic perturbations tend to affect drug interactions. If could be interesting to study the mutations found in those studies to see if they support some of the conclusions of this paper (e.g. is there any overlap between the genes mutated in the earlier studies and the knock outs found to perturb drug interactions in this study).

We agree with the reviewer that both papers are relevant for this part of the introduction. We added references to them and revised the introduction accordingly (p. 3, paragraph 2). As suggested by the reviewer, we checked the mutations identified in the evolved strains reported in (Munck et al. 2014). Only two drug combinations were investigated in both (Munck et al. 2014) and in our study: ciprofloxacin-tetracycline and chloramphenicol-tetracycline (no drug pairs overlap between (Evgrafov et al. 2015) and our study). For ciprofloxacin-tetracycline, Munck et al. reported point mutations or indels in *envZ, puuB, marR, gyrA, ompF, rpoC, soxR, thrA*, and *rob*. We identified none of these genes as altering the drug interaction in our study. (This can have various reasons: e.g. combinations of these mutations may be necessary to alter the drug interaction; the mutations in Munck et al. are also different from gene deletions and it would be important to measure the entire response surface of these mutants for a thorough comparison with our results.) Intriguingly, Munck et al. found that the chloramphenicol-tetracycline interaction changed relatively little in most resistant mutants, consistent with the high robustness of this drug interaction to genetic perturbations we observed (Figure S3). We have added a sentence mentioning this observation where we describe the unusual robustness of the chloramphenicol-tetracycline interaction (p. 6, paragraph 1).

(2) Also, the work of the Miller lab should be acknowledged (Liu et.al. 2010) in the discussion of the initial characterization of the KEIO collection. It would also be nice to cross validate the findings of Liu et.al. with the results of this study in the supplement. E.g. look at the overlap between the changes in susceptibility found previously for the KEIO collection with the changes in growth rate found in the present study.

We have followed this suggestion and added a reference to the Liu et al. paper (p. 4, paragraph 2). To cross-validate our measurements with these previous results, we compared the data for all antibiotics that overlap between our study and (Liu et.al. 2010); these are ciprofloxacin, tetracycline, chloramphenicol, and nitrofurantoin. Specifically, for each of these drugs, we used the lists of the gene deletion strains that were reported to be sensitive in the supplemental material of (Liu et al. 2010) and determined the corresponding sensitivity rank of these strains in our response data; the sensitivity rank of each strain was obtained by sorting the deletion strains in order of decreasing sensitivity to the drugs based on our growth response measurements. In all cases, the vast majority of strains identified as sensitive in (Liu et al. 2010) are also among the most sensitive ones in our data set. As suggested by the reviewer, we have added a new supplemental figure (S10) that shows these cross-validation results.

(3) It appears from figure 2D that the tendency is towards antagonism for the genetic perturbation outliers. In Figure S4, there appears to be clear trends in either direction for each combination (also towads synergism in Fig S5 F). It would be beneficial to discuss this in the manuscript and possibly its relation to the WT interactions. The authors have already some discussion, but I would suggest that this is expanded.

We agree that these tendencies for drug interactions to change in a certain direction are interesting, in particular because they may indicate in which direction a drug interaction is likely to change during the evolution of drug-resistance or in different strains of the same bacterial species. We have expanded the explanation of this effect in the main text (p. 6, paragraph 1) where we describe the two drug pairs that showed clear biases and explicitly mention that these biases occur in opposite directions even for very similar wild type drug interaction. In the same paragraph, we now explicitly mention that other drug pairs do not show such clear global biases (Fig. S5). We have further added a sentence to the discussion of this point at the end of the main text (p. 9, paragraph 2) where we explain that such biases in drug interaction changes are relevant for the design of drug combinations but that their causes are unknown.

Reviewer #3:

General Summary

The manuscript "Systematic discovery of drug interaction mechanisms" by Chevereau and Bollenbach is exciting primarily because of the new finding that the detailed 'shape' of drug interactions is generally preserved across diverse genetic perturbations, even where those perturbations substantially alter sensitivity of the organism to one drug or another. This is exciting because a single detailed measurement of drug interactions in one genetic background can then be 'warped' to predict the effects in other genetic backgrounds. Exceptions to this newly established phenomenon are therefore also interesting, and some of these are described and explored. The experiments seem generally well designed, and it was an impressive amount of work to phenotype individual strains as opposed to competitively-grown barcoded populations.

The follow-up studies are useful, and acceptably round out the contribution of the large-scale studies. However the two most fleshed out examples do not really constitute "systematic discovery of drug interaction mechanisms" that would support the manuscript's title. However, with a title that better captured the main contribution (highly predictive modeling of drug x drug x gene interactions from drug x drug interactions), publication of this study would represent a very useful contribution to the field.

We thank the reviewer for his/her thorough evaluation and appreciation of our work. We also appreciate the suggestion to change the title and agree that alternative titles could capture key results of our manuscript. While we have carefully considered such alternatives, we feel that the follow-up experiments we performed, while limited to two main specific drug combinations, support that our approach enables

the systematic discovery of drug interaction mechanisms based on the genes and cellular functions we identified. Hence, we would prefer to leave the title as is.

There were some issues with clarity about how the drug/drug/gene trios were chosen for the detailed measurements of drug interaction. I'll summarize what I think was done overall and refer back to this below:

-*Expt 1: 6 drug x genome experiments*
-*Expt 2: (drug A + drug B) x genome for 6 out of the 15 possible pairwise drug combinations*
-*Expt 3: 108 drug x drug experiments in particular gene mutant backgrounds*
-*Various followup experiments*

Major Issues

-*It was not at all clear how the 108 drug x drug x gene experiments were chosen. Was this based on Expt 2? If so how? Any biases in the selection process of 108 experiments could affect the downstream conclusions.*

We thank the reviewer for pointing out this issue (which is closely related to the reviewer's 2nd point under "minor issues" below). Indeed, we chose the 108 drug x drug x gene experiments based on data from Expt 2. For each drug pair, we compared the observed growth rate from Expt 2 to the expected growth rate which was calculated from Expt 1 (as shown in Fig. 2D;3A,B;S5); we then selected 18 genes that covered (a) the most significant outliers (for which observed and expected growth rate strongly differed) and (b) additional genes that led to a clear change in sensitivity to at least one of the constituent drugs. The purpose of Expt 3 was to validate unequivocally that the outliers indeed have altered drug interactions (Fig. 3C;S6) and to challenge the general principle shown in Fig. 2A-C in the strongest possible way (this principle also holds for gene deletion mutants with unchanged sensitivity to the drugs but this is a much weaker result because the entire growth response to the drug combination is typically just identical to that of the wild type for such mutants). To clarify this issue and the role of Expt 2, we added a sentence explaining the selection of these strains in the main part describing the validation of the general principle (p. 5, paragraph 1) and another sentence in the Materials and Methods at the beginning of section "Two drug response surfaces" (p. 11, paragraph 2). We have further revised the explanation of our strategy for identifying candidate genes that affect drug interactions (p. 6, paragraph 1; see also response to the 2nd point under "minor issues" below).

-*"...the ATP synthase mutant atpF was more sensitive to trimethoprim and this sensitivity was reduced by chloramphenicol or mecillinam, leading to suppression (Fig. 3C); a similar effect occurred for ciprofloxacin-tetracycline (Fig. S6S). As DNA repair and synthesis require ATP (Waldstein et al., 1974), these observations suggest that the repair of DNA damage (caused by trimethoprim or ciprofloxacin) is impaired by low intracellular ATP concentration in the mutant; inhibiting protein synthesis should alleviate this impairment and increase growth as it reduces ATP turnover and increases intracellular ATP concentration (Schneider et al., 2002)."*

The connection of ATP synthase with DNA repair is a reasonable speculation, but a great many cellular processes require ATP, and some subset of these (e.g., nucleotide biosynthesis, replication) will impact DNA repair indirectly. The observation that a phenotype depends on ATP does not point any more to DNA repair than it does to any other ATP-requiring process, so it cannot be claimed from this evidence that we now understand the mechanistic effects of atpF mutation on drug interaction. Other lines of evidence pointing to ATP level effects as the cause of changing drug interaction do not resolve this issue.

Moreover, even if we believe that lowering ATP levels inhibits DNA repair, it is not clear why oligomycin would change the nature of the chloramphenicol-trimethoprim interaction from antagonistic to suppressive, so can we say that we understand the mechanism of drug interaction.

Whether or not the interaction mechanisms are understood does not detract from what I saw as the main message of the paper, but if the authors want to claim they are revealing mechanisms, they should provide better arguments for this.

We thank the reviewer for pointing out that this scenario was not clearly explained. We agree that changes in ATP concentration can affect diverse cellular processes. While we provide several lines of evidence that the observed drug interaction change is caused by changes in ATP synthesis (which suggests a mechanism at the cellular level) we agree that our data do not prove the molecular mechanism linking ATP and DNA repair. We still wanted to suggest this as a plausible scenario at the molecular level. While this scenario is slightly involved, we do think that it offers a plausible explanation for the change of drug interaction from antagonistic to suppressive. We have entirely rewritten the paragraph describing this scenario (p. 8, paragraph 2) and, in particular, separated the description of the general role of ATP in this drug interaction clearly from the more speculative part about the molecular link between ATP and DNA repair; the latter is now mentioned only in a single sentence near the end of the paragraph. In addition, we now describe this molecular scenario in more cautious terms and state explicitly that changes in ATP synthesis could affect many other cellular processes.

Minor Issues

-For Expt 2, the authors used a single mix of drug A and drug B at the same concentrations used in the single-drug experiments. The main text will be interpreted by most to mean that they diluted the drug combo to get 30% inhibition, but then the Materials and Methods says that they only diluted the drug combo for one pair that was dead, and otherwise used the same concentrations used for the single agents. Thus, the % inhibition must be generally higher than 30% for the drug combo experiments, but this is not discussed and % inhibitions are not provided.

To clarify this point, we added a sentence and revised the corresponding part in the first paragraph of the "Results and discussion" section (p. 4, paragraph 2). We further added a sentence to the methods part (p. 10, paragraph 1) where we now state the relative growth inhibitions under all drug combinations.

-More importantly, Expt 2 seems irrelevant to the paper... I did not see any place where it was used outside of Fig 1F, and it was not clear what was learned from it there. If it wasn't used, this is not a major issue, and certainly the data should be reported either way, but if the authors intended to draw any conclusions from this experiment it should be made more clear. (Was it used to choose the 108 tests in Expt 3? See above.) At the very least the authors should tell us whether Expt 3 results agreed with Expt 2 results.

Expt 2 served two main purposes: (i) to validate the principle that enables the prediction of mutant growth rates under drug combinations from their growth rates under the constituent drugs genome-wide (Fig. 2D;S5) and (ii) to make an informed decision about the 108 tests in Expt 3 (see also our response to the first point under “major issues” above). Apart from Fig. 1F, data from this experiment are shown in Fig. 2D, 3A,B, S2, and S5. Expt 2 is crucial for our analysis as it is needed to identify outlier genes that change drug interactions. The results of Expt 3 (in which these outliers were verified) agreed well with those of Expt 2; this is shown in Fig. 3 where panels A and B show the outlier mutants identified in Expt 2 and panel C shows results of Expt 3 which confirm that the drug interaction changed in these mutants as expected from the result of Expt 2. To clarify the role of this experiment, we now state explicitly in the main text that the outliers were identified based on significant deviation between the observed growth rate from Expt 2 and the expected growth rate from Expt 1 (p. 6, paragraph 1). We have further added a

sentence to the legend of Fig. 3 which explicitly mentions the agreement between the results of Expt 3 with those of Expt 2.

-Were there any replicates amongst the 108 drug x drug x gene experiments? Some sense of reproducibility of these studies should be provided?

Yes, we validated the key effects in replicate experiments. Specifically, for each drug pair, we measured the response surface of the wild type and of mutants that showed a clear change in drug interaction (Fig. 3C;S7) at least in duplicate. Replicate response surfaces measured on different days were generally highly reproducible (Fig. S1) and, in particular, all drug interaction changes in mutants were confirmed. This high reproducibility is likely due to the automation of our assay and the intrinsic redundancy of these two-dimensional concentration gradient experiments which are done at fine concentration resolution: the drug concentrations in neighboring wells are quite similar and serve as an internal consistency check -- any measurement errors or fluctuations from individual wells are immediately noticeable and quite rare. We now comment in more detail on replicates and the reproducibility of our measurements at the end of the section "Two drug response surfaces" of the Materials and Methods part (p. 12, paragraph 1).

-*"Synergism and antagonism occur frequently between antimicrobials and are largely determined by the primary cellular target of the drugs that are combined (Cokol et al., 2011; Ocampo et al., 2014; Yeh et al., 2006)" While Ocampo et al and Yeh et al support this statement, the results of Cokol et al do not. In Cokol et al, most synergistic interactions amongst pairs of drugs with 'known targets' did not correspond to target pairs encoded by genes with negative genetic interactions. Drug interaction may be largely determined by the primary cellular target; however, results of Cokol et al suggest that for this to be true, drugs must generally target more and/or different gene products than those that are currently known.*

We thank the reviewer for pointing out this issue. To clarify this point, we have removed the (Cokol et al, 2011) reference from this sentence in the introduction and added a new sentence explaining this result of the Cokol et al. paper (p. 3, paragraph 1).

-*" Further, ATP synthase expression increased two-fold in response to trimethoprim (Fig. S8), suggesting that cells counteract ATP deficiency" Maybe "respond homeostatically to" would be more accurate than "counteract" as no evidence is presented that the deficiency has actually been remediated by increased ATP synthase expression.*

We have followed this suggestion and changed the corresponding sentence accordingly (p. 8, paragraph 2).

-*"Indeed, inhibiting ATP synthase led to suppression between trimethoprim and chloramphenicol..." Were oligomycin and venturicidin synergistic with trimethoprim?*

We did not find any indication for synergism between trimethoprim and oligomycin (or venturicidin). A technical problem here is that oligomycin and venturicidin are poor antibiotics and consequently had little effect on growth even at the highest concentrations we could achieve. Hence, we cannot determine these drug interactions rigorously by measuring the complete response surface as we did for antibiotic pairs throughout the manuscript. (An additional practical problem is that oligomycin and venturicidin are substantially more expensive than other drugs used in our study which effectively prohibits their use in large-scale experiments.) However, over the concentration range that we could cover in our experiments, oligomycin and venturicidin had little effect on trimethoprim sensitivity, suggesting that there is no strong synergism. We have added a sentence in the legend of Fig. S9 where we mention this observation. Note that the scenario above and our main conclusions do not predict or require synergism (or any other

particular interaction) between these drugs: we simply use oligomycin and venturicidin to inhibit ATP synthase.

- *It would have been interesting to measure EDTA interaction with nitrofurantoin*

We agree that this would be interesting but, similar to oligomycin and venturicidin, EDTA does not substantially inhibit growth at the concentrations used in our experiments. We further noticed that EDTA has complicated, non-monotonous effects on the final growth yield at higher concentrations (potentially because it has non-specific effects other than chelating metal ions at high concentrations). Hence, we cannot rigorously characterize the interaction between EDTA and antibiotics from the response surface as we did for antibiotic pairs. Still, the data in Fig. 4A,B indicate strong synergism between EDTA and chloramphenicol (as sensitivity to chloramphenicol increases considerably even at EDTA concentrations that have little effect on growth, see shift of IC50 and MIC line on the y-axis between Fig. 4A and B); this effect supports the scenario for the underlying mechanism of this drug interaction discussed in the main text. Similarly, Fig. 4A,B shows that EDTA leads only to a slight increase in sensitivity to nitrofurantoin. We now explicitly mention these effects of EDTA on antibiotic sensitivity in the legend of Fig. 4.

Acceptance letter 15 April 2015

Thank you again for sending us your revised manuscript. We are now satisfied with the modifications made and I am pleased to inform you that your paper has been accepted for publication.

© European Molecular Biology Organization
